# Supplementary material for: Determining the global citizenship tendencies of international university students for a sustainable world
Source: PLoS One. 2025 Oct 31;20(10):e0335817. doi: 10.1371/journal.pone.0335817 (PMC12578196; doi:10.1371/journal.pone.0335817)
Supplement: Attachment — Submitted filename: PONE-D-25-07333_reviewer.pdf [file pone.0335817.s003.pdf]

PLOS ONE

Determining the Global Citizenship Tendencies of International University Students for a Sustainable World

--Manuscript Draft--

**Manuscript Number:**	PONE-D-25-07333
**Article Type:**	Research Article
**Full Title:**	Determining the Global Citizenship Tendencies of International University Students for a Sustainable World
**Short Title:**	International Students' Global Citizenship and Sustainability
**Corresponding Author:**	OSMAN AKHAN<br>Akdeniz University: Akdeniz Universitesi<br>KONYAALTI, ANTALYA TÜRKIYE
**Keywords:**	Global citizen; Sustainability; international students
**Abstract:**	This study examines the global citizenship tendencies of international university students and the effects of these tendencies on a sustainable world. In this context, 634 international university students studying in Turkey were determined as participants. In the study conducted with mixed methods, quantitative data were collected with the Global Citizenship Scale, and qualitative data were collected through face-to-face interviews with the students. When the results of the research are evaluated overall, the quantitative findings describe global citizenship awareness at a moderate level, while the qualitative findings indicate that this awareness is higher and that students can assess this concept in a multifaceted manner. Both quantitative and qualitative findings coincide in that students develop sensitivity to environmental and social problems and that the active participation of individuals increases global citizenship awareness. In addition, it was determined that the participants reflected global citizenship awareness in their daily lives and adopted actions such as sustainable consumption and resource management. The study emphasizes that international students have a critical role in developing sustainability and global awareness and offers suggestions for educational policies.
**Order of Authors:**	Adem Uzun, PhD
	Osman Akhan, Associate Professor
	Saim Turan, PhD Candidate
	Ali Özkaya, Associate Professor
**Additional Information:**	
**Question**	**Response**
**Financial Disclosure**<br><br>Enter a financial disclosure statement that describes the sources of funding for the work included in this submission. Review the submission guidelines for detailed requirements. View published research articles from *PLOS ONE* for specific examples.<br><br>This statement is required for submission and **will appear in the published article** if the submission is accepted. Please make sure it is accurate.	The author(s) received no specific funding for this work.

Powered by Editorial Manager® and ProduXion Manager® from Aries Systems Corporation

**Funded studies**<br>Enter a statement with the following details:<br>• Initials of the authors who received each award<br>• Grant numbers awarded to each author<br>• The full name of each funder<br>• URL of each funder website<br>• Did the sponsors or funders play any role in the study design, data collection and analysis, decision to publish, or preparation of the manuscript?<br><br>Did you receive funding for this work?	
**Competing Interests**<br><br>Use the instructions below to enter a competing interest statement for this submission. On behalf of all authors, disclose any competing interests that could be perceived to bias this work—acknowledging all financial support and any other relevant financial or non-financial competing interests.<br><br>This statement is required for submission and **will appear in the published article** if the submission is accepted. Please make sure it is accurate and that any funding sources listed in your Funding Information later in the submission form are also declared in your Financial Disclosure statement.<br><br>View published research articles from *PLOS ONE* for specific examples.	The authors have declared that no competing interests exist.

**NO authors have competing interests**<br><br>Enter: *The authors have declared that no competing interests exist.*<br><br>**Authors with competing interests**<br><br>Enter competing interest details beginning with this statement:<br><br>*I have read the journal's policy and the authors of this manuscript have the following competing interests: [insert competing interests here]*<br><br>* typeset	
**Ethics Statement**<br><br>Enter an ethics statement for this submission. This statement is required if the study involved:<br><br>• Human participants<br>• Human specimens or tissue<br>• Vertebrate animals or cephalopods<br>• Vertebrate embryos or tissues<br>• Field research<br><br>Write "N/A" if the submission does not require an ethics statement.<br><br>General guidance is provided below. Consult the submission guidelines for detailed instructions. **Make sure that all information entered here is included in the Methods section of the manuscript.**	Studies involving humans were approved by the Akdeniz University Social and Human Sciences Ethics Committee Decision. 19 October 2024 No: 395. The studies were conducted in accordance with local legislation and institutional requirements. Participants gave their written informed consent to participate in this study.

Powered by Editorial Manager® and ProduXion Manager® from Aries Systems Corporation

**Format for specific study types**<br><br>**Human Subject Research (involving human participants and/or tissue)**<br>• Give the name of the institutional review board or ethics committee that approved the study<br>• Include the approval number and/or a statement indicating approval of this research<br>• Indicate the form of consent obtained (written/oral) or the reason that consent was not obtained (e.g. the data were analyzed anonymously)<br><br>**Animal Research (involving vertebrate animals, embryos or tissues)**<br>• Provide the name of the Institutional Animal Care and Use Committee (IACUC) or other relevant ethics board that reviewed the study protocol, and indicate whether they approved this research or granted a formal waiver of ethical approval<br>• Include an approval number if one was obtained<br>• If the study involved *non-human primates*, add *additional details* about animal welfare and steps taken to ameliorate suffering<br>• If anesthesia, euthanasia, or any kind of animal sacrifice is part of the study, include briefly which substances and/or methods were applied<br><br>**Field Research**<br><br>Include the following details if this study involves the collection of plant, animal, or other materials from a natural setting:<br>• Field permit number<br>• Name of the institution or relevant body that granted permission	
**Data Availability**<br><br>Authors are required to make all data underlying the findings described fully available, without restriction, and from the time of publication. PLOS allows rare exceptions to address legal and ethical concerns. See the PLOS Data Policy and FAQ for detailed information.	No - some restrictions will apply

A Data Availability Statement describing where the data can be found is required at submission. Your answers to this question constitute the Data Availability Statement and **will be published in the article**, if accepted.<br><br>**Important:** Stating 'data available on request from the author' is not sufficient. If your data are only available upon request, select 'No' for the first question and explain your exceptional situation in the text box.<br><br>Do the authors confirm that all data underlying the findings described in their manuscript are fully available without restriction?	
**Describe where the data may be found in full sentences. If you are copying our sample text, replace any instances of XXX with the appropriate details.**<br><br>• If the data are **held or will be held in a public repository**, include URLs, accession numbers or DOIs. If this information will only be available after acceptance, indicate this by ticking the box below. For example: *All XXX files are available from the XXX database (accession number(s) XXX, XXX.*).<br>• If the data are all contained **within the manuscript and/or Supporting Information files**, enter the following: *All relevant data are within the manuscript and its Supporting Information files.*<br>• If neither of these applies but you are able to provide **details of access elsewhere**, with or without limitations, please do so. For example:<br><br>*Data cannot be shared publicly because of [XXX]. Data are available from the XXX Institutional Data Access / Ethics Committee (contact via XXX) for researchers who meet the criteria for access to confidential data.*<br><br>*The data underlying the results presented in the study are available from (include the name of the third party*	Data cannot be shared publicly because of Ethics. Data are available from the Akdeniz University Institutional Data Access / Ethics Committee (contact via mail to the corresponding author) for researchers who meet the criteria for access to confidential data.

Powered by Editorial Manager® and ProduXion Manager® from Aries Systems Corporation

and contact information or URL).<br>• This text is appropriate if the data are owned by a third party and authors do not have permission to share the data.<br><br>* typeset	
Additional data availability information:	

Powered by Editorial Manager® and ProduXion Manager® from Aries Systems Corporation

Manuscript

Click here to access/download;Manuscript;PLOSONE candiadate article.docx

Determining The Global Citizenship Tendencies of International University Students for a Sustainable World

Language & Style:
- Grammar and sentence structure need revision throughout for clarity.
- Several long or complex sentences would benefit from simplification.

Adem Uzun[1], Osman Akhan*[2], Saim Turan[3] and Ali Öz[illegible]

[1]Department of Gifted Education, Faculty of Education, Cumhuriyet U[illegible] Turkey

[2]Department of Social Studies Education, Faculty of Education, Akdeniz University, Antalya 07000, Turkey

[3]Department of Social Studies Education, Faculty of Education, Akdeniz University, Antalya 07000, Turkey

[4]Department of Mathematics and Science Education, Faculty of Education, Akdeniz University, Antalya 07000, Turkey

* Corresponding author

E-mail: osmanakhan07@gmail.com

- Revise abstract for clarity and conciseness.
- Add key quantitative and qualitative results

Abstract

This study examines the global citizenship tendencies of international university students and the effects of these tendencies on a sustainable world. In this context, 634 international university students studying in Turkey were determined as participants. In the study conducted with mixed methods, quantitative data were collected with the Global Citizenship Scale, and qualitative data were collected through face-to-face interviews with the students. When the results of the research are evaluated overall, the quantitative findings describe global citizenship awareness at a moderate level, while the qualitative findings indicate that this awareness is higher and that students can assess this concept in a multifaceted manner. Both quantitative and qualitative findings coincide in that students develop sensitivity to environmental and social problems and that the active participation of individuals increases

global citizenship awareness. In addition, it was determined that the participants reflected global citizenship awareness in their daily lives and adopted actions such as sustainable consumption and resource management. The study emphasizes that international students have a critical role in developing sustainability and global awareness and offers suggestions for educational policies.

Keywords: Global citizen, sustainability, international students

Introduction

- The rationale behind the study is not clearly stated.
- Prior literature is insufficiently reviewed; consider adding 2–3 key references.

Since the second ha [illegible] a period of rapid change in political, economic, social and cultural areas, especially with the impact of developments in information and communication technologies. With this process of change, inequality, poverty and human rights violations have become threats to existing peace and environmental sustainability in the world that has become an interconnected/dependent structure. In response to these challenges, the concept of global citizenship has been defined as a way out for an alternative, inclusive and sustainable world.

Sustainable development is an important movement that initiated from the idea that every individual living in the world should have the best living conditions. Therefore, it requires a worldwide effort because the problems addressed are not just the problems of one country, but the problems of the entire world. At this point, if global citizens act with a common understanding to produce solutions to these problems, a more balanced sustainable development process can be achieved. Global citizens learn to work together for a more sustainable future, equal rights and opportunities not only for their own countries, but for the entire world. When sustainable development is supported by the understanding of global citizenship, a more fair, equal and environmentally friendly world becomes possible. Thus, global citizenship strengthens the social dimension of sustainable development.

The concept of global citizenship must first be developed in the minds of young people and established on a solid foundation because young people are the leaders, decision-makers and pioneers of social change of the future. Therefore, developing the concept of global citizenship in young people is a critical step. When young people are raised with the idea of global citizenship, they can be pioneers of positive change not only in their own countries but also in the whole world. At this point, international students who take the first step towards global citizenship by studying in different countries play an important role in increasing cultural awareness and empathy. To build a more peaceful, fair and sustainable world for all humanity, these students encourage respect for different cultures, traditions and lifestyles around the world.

Globalization and the Concept of Global Citizenship

Today, the adjective global is frequently used in a variety of areas, such as global climate change, global epidemics, global economic crises, global trade, global governance, and global security because the sphere, where interaction takes place, has become a place where problems and solutions concern the whole. The concept of globalization, which first emerged in the 1960s, began to be frequently used after the 1980s [1]. By the 1990s, it had become a key word accepted by scientific circles [2].

The phenomenon of globalization is a multifaceted and complex concept that can be explained socially, politically, technologically and economically [3]. This situation has led to many definitions of globalization in the literature. Robertson [4] expresses globalization as the shrinking of the world and at the same time the strengthening of world consciousness. Akhan and Kaymak [5] defined globalization as a living and renewable structure that includes all aspects of intellectual, economic, cultural, and technological changes and has the ability to affect human social life.

Globalization is the process of economic, political and cultural integration of the world [6]. Globalization combines economic, social and environmental benefits, and these benefits have transformed, diversified and shaped communities around the world in ways that keep them connected despite being physically distant from each other [7].

What does it mean to be a global citizen? Is it just about knowing the capitals of countries, discovering famous landmarks, tasting famous cuisines, or being informed about world events? Or does it go beyond knowledge to take action that contributes to a better, more sustainable future? In today's interconnected world, global citizenship is not just about awareness; it is also a commitment to promoting positive change across borders [8].

Important international organizations working on issues such as social development, human rights and sustainability around the world have emphasized the following topics for global citizenship [9–11]:

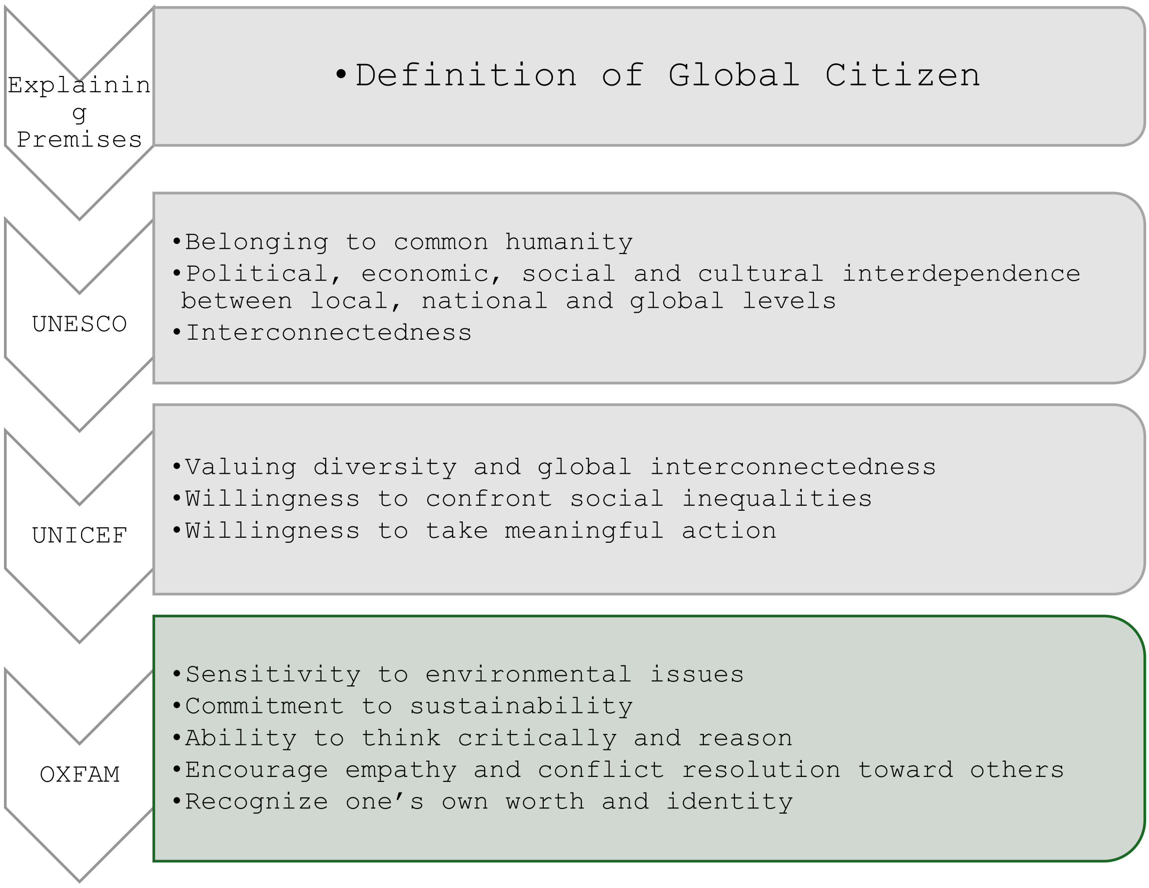

Figure 1. Global Citizen's Explanation

Looking at Figure 1, we can say that leading organizations, each of which is trying to create a more fair and equal society in the world with different focus areas and generally addressing the same social problems and seeking solutions to these problems, expect global citizens to act with a sense of social responsibility and contribute to creating positive changes around the world.

Traditional concept of citizenship has been transformed by globalization, international treaties and agreements, and international human rights protection frameworks. In addition, the expansion of information and communication technologies has facilitated the formation of international networks and communities that share common interests and concerns [12]. This has strengthened the sense of belonging to a global community and created a sense of world citizenship identity and civic participation in global issues. Increasingly diverse societies have

also shaped this transformation. The traditional national citizenship model no longer reflects today's changing realities [13]. At this point, based on the fact that individuals are part of a community living together in a global world, global citizenship is a concept of citizenship that requires individuals to be sensitive to global issues as well as their responsibilities towards their own countries [14].

Today, the rapid transformation of local problems into global issues increases the importance of individuals acting with this common consciousness. For example, the COVID-19 pandemic, which emerged in Wuhan, quickly turned into a global health crisis and affected the entire world [15,16]. The Syrian civil war, which began in the 2010s, led to a refugee crisis and triggered the problem of international migration [17]. Again, climate change is making its effects felt worldwide, from the melting of Greenland glaciers to forest fires, and as a problem that threatens global ecosystems, economies, and societies, it affects all societies living on earth [18–21].

To solve these problems for the benefit of humanity and to have a more liveable world, it is necessary to focus not only on the awareness that "comes with global citizenship" but also on how the solutions should be shaped; this can be explained by the concept of sustainability. In today's world where the problems that humanity faces transcend national borders, sustainability is at the center of citizenship [22]. As the challenges we face increasingly transcend national borders, the search for sustainability emerges as a vital framework to promote global cooperation and ensure a liveable future for everyone [23]. Thus, in its 2030 agenda, the United Nations aims to develop intercultural understanding, tolerance, mutual respect and an ethic of shared responsibility through global citizenship; by acknowledging the natural and cultural diversity of the world, it has stated that "all cultures and civilizations can contribute to sustainable development and are an important supporter of this" [24].

The Relationship Between Sustainability and Global Citizenship

Sustainability, whose basic principle is to oppose monopolization and individualization, essentially represents the state or quality of something that can be sustained and continued. Therefore, sustainability has ceased to be a modern environmental movement and has become a way of thinking and acting that focuses on the cultures of societies. As a way of thinking, sustainability is an approach that represents overcoming the differences between societies and living together in a desired future [25].

Sustainable development, on the other hand, advocates addressing the cultures of all societies and acting according to their needs, as it is a social activity plan. At the same time, sustainable development, which aims to provide humanity with better living conditions and increase its welfare, has become a central field of study since it focuses on political concepts such as freedom, social justice, rights, law, etc. [26]. In addition, sustainable development has emerged as universal goals that aim to meet the needs of today's generations while also considering the needs of future generations, combating inequalities, recognizing the importance of cooperation, and supporting societies [27]. These goals are the Sustainable Development Goals, consisting of 17 main goals and 169 sub-goals [28].

The Sustainable Development Goals explain the concept of global citizenship, especially in the context of Goal 16, Peace, Justice and Strong Institutions, with the aim of establishing justice, peace, equality, human rights and strong institutions. In addition, Goal 4, Quality Education, also tries to spread the concept of global citizenship. Here, individuals are encouraged to be informed about global issues, to respect other cultures and to be active and responsible individuals on a global scale through education. Individuals who are sensitive to global issues, can communicate with different cultures and have a multi-faceted perspective can be leaders who can transform societies. In this context, international students have significant potential.

As countries wage war, exchange programs provide opportunities for young people to make friends with people from other parts of the world. Furthermore, the increasingly globalized economy will require more of the future workforce to have the skills to effectively interact with those from other cultures [29]. Developing global citizenship can be achieved through educational experiences gained by living abroad [30]. Students who study abroad are reported to be willing to change their perspectives on global issues and gain from their international experiences in terms of their ability to adapt to new situations and communicate effectively [31]. Gibson, Rimmington, and Landwehr-Brown [32] stated that successful intercultural collaboration and effective communication between people from different cultures can create a greater sense of global awareness.

Since the problems the world is facing today, from climate change to social inequality, require solutions that transcend national borders, it is important to understand the global citizenship understanding of international university students through different tools. Indeed, since international students interact with different cultures, they gain experience in issues such as global citizenship, intercultural interaction, and the impact of local problems on people and societies in different parts of the world. Indeed, international students who develop an understanding of sustainability that they will carry into their lives through their education and interactions become important actors for a sustainable future with the different perspectives and experiences they acquire [33]. This study aims to provide valuable information on how international students perceive and implement global citizenship by investigating the global citizenship tendencies of international university students, their contributions to sustainable global solutions, students' awareness, attitudes and behaviours on global citizenship and sustainability, their empathy towards other cultures and their sense of responsibility regarding sustainability. It is hoped that the research findings will inform educational policies and

programs that aim to raise global citizens who can contribute to a sustainable and interconnected world.

Method

- Description of participants and data collection lacks detail.
- Statistical methods need clarification, particularly around model assumptions and validity checks.

Research Model

This study used a mixed method, which uses both quantitative and qualitative data collection and analysis. Quantitative and qualitative methods each provides different types of data and accesses different aspects of the phenomenon being studied. Thus, by integrating qualitative and qualitative findings, the scope, density, detail and even validity of the study are increased [34]. Mixed designs can be preferred in educational sciences and social science research when in-depth information is needed, to use quantitative and qualitative data together [35].

The quantitative and qualitative data obtained in this study were collected simultaneously and completed in a single stage. In this respect, the research was designed as a convergent parallel mixed design. The quantitative and qualitative data obtained in this study were given equal importance in terms of importance [35]. In the convergent parallel mixed research design, quantitative and qualitative data are collected simultaneously, analyses are made separately, and the findings are integrated and interpreted [36].

Study Group

The study group of this research consists of 634 international students (323 females, 311 male) studying at four different universities in Turkey. While selecting the students, the conditions were taken into consideration that they had completed at least one semester of education in Turkey with international student status and had English language proficiency. Of the 634 international university students determined as the study group of the research, 60 stated that they came from Asia, 154 from Europe, 118 from Russia/Eurasia, 103 from Sub-Saharan Africa,

and 199 from the Middle East and North Africa region. These students were reached by obtaining their phone and e-mail addresses through the international relations offices of their universities.

The data collection stage and ethical procedure

First, students who met the conditions were sent an e-mail and the subject of the study and what was asked of them in the study were mentioned. Students who did not respond to the e-mail were tried to be reached again via the WhatsApp application on their phones. Then, the Global Citizenship Scale, which constitutes the quantitative dimension of the study, was sent as a Google form to students who wanted to participate in the study via e-mail / WhatsApp. At the end of the relevant form, the question "Would you like to contribute to this study by conducting a face-to-face interview?" was asked. There were 43 volunteer students who answered yes to this question and agreed to spend time on the study. Students were reminded to complete the scale sent via Google form before coming to the interview. 6 students could not participate in the interview for various reasons (illness, giving up, etc.), and the remaining 37 students were accepted as the study group for the qualitative dimension of the research.

Participants were informed about the research in the Google Form and their consent was obtained. The data were collected between October 15, 2024, and December 25, 2024. This study is approved by Social Sciences and Humanities Scientific Research and Publication Ethics Committee of Akdeniz University.

The data of this study was collected with a scale in the quantitative dimension and with the interview method in the qualitative dimension. In the quantitative dimension of the study, after obtaining the necessary permissions, the "Global Citizen Scale" developed by Reysen and Katzarska-Miller [37] was used. The scale was delivered to the students in the study group by

creating a Google Form via e-mail / WhatsApp. The original language of the scale is English. Since the students in the study group were selected from people who spoke English, the scale could be applied in its original language, therefore no language validity process was applied. The Global Citizen Scale was developed with a total of 22 items, 9 factors, and a 7-point Likert type. The items were graded between "Strongly Agree (7)" and "Strongly Disagree (1)" and the scale was structured in a way that a maximum of 154 points and a minimum of 22 points could be obtained. Items 1, 2, 3 and 4 of the scale represent the "Normative Environment" factor, items 5, 6, 7 and 8 represent the "Global Awareness" factor, items 9 and 10 represent the "Global Citizenship Identification" factor, items 11 and 12 represent the "Social Justice" factor, items 13 and 14 represent the "Valuing Diversity" factor, items 15 and 16 represent the "Intergroup Empathy" factor, items 17 and 18 represent the "Environmental Sustainability" factor, items 19 and 20 represent the "Intergroup Helping" factor, and items 21 and 22 represent the "Responsibility to Act" factor. The internal consistency coefficients of the sub-factors constituting the scale are Normative Environment (.82), Global Awareness (.80), Global Citizenship Identification (.89), Intergroup Empathy (.76), Valuing Diversity (.91), Social Justice (.74), Environmental Sustainability (.76), Intergroup Helping (.76) and Responsibility to Act (.78).

For the qualitative dimension of the study, 9 interview questions were prepared by the researchers. While creating the interview questions, a series of questions that would reveal the participant perceptions directly related to the Global Citizen Scale developed by Reysen and Katzarska-Miller [37] were first developed. After the interview questions were created, expert opinions were obtained from two faculty members who were knowledgeable about global citizenship. The interview form created after receiving the expert opinion was applied to 5 international university students who were not in the study group and the points they did not understand were determined and the interview form was revised accordingly. The finalized

interview form was applied to 37 international university students determined as the study group by face-to-face interviews. For the interviews, the students were contacted with an appointment using the WhatsApp application, and the place, day and time of the interview were determined. The interviews were conducted at the faculty where the students were studying. The interviews were attended by two researchers and the answers given were both audio-recorded and notes were taken by the researchers. After the interviews, the interviews were transcribed and made ready for data analysis.

Analysis of Data

The quantitative data set of the study was analyzed with SPSS 22. The Jamovi [38] program was used to evaluate the normality assumption and to carry out the analysis process. To evaluate the normality of the scores, the central tendency measures of the participant scores were examined and it was seen that they were relatively close to each other. If the skewness and kurtosis values are between -1 and +1, the distribution shows normal distribution characteristics [39]. Tabachnick and Fidell [40] stated that normal distribution is provided if this value is between -1.5 and +1.5. When the skewness and kurtosis values of the scores belonging to the subgroups of demographic variables are examined, it is seen that they are between the tolerance values. In addition, the Shapiro Wilk test was performed to examine whether the scores exhibited a normal distribution and normality was examined. As a result of the analysis, normality was provided for the 18-20 age ideal environment score, 18-20 age global awareness score and 18-20 age total score, $p>0.05$; It was observed that normality was not provided for other scores, $p<0.05$. Since the normality assumption was not provided for the social media score and scale scores, the analysis process was carried out with Spearman-Brown Correlation. The Mann Whitney U Test was used to examine whether there was a significant difference for the variables of gender, participation in global events and association membership. The biserial

correlation coefficient was calculated as the effect size for the Mann Whitney U Test [41]. A coefficient of 0.1 indicates small, 0.3 indicates medium and 0.5 indicates large effect size. The Kruskal Wallis H Test was used to determine whether the difference between the scale scores for the country and age variables was significant. Epsilon square was calculated as the effect size for the Kruskal-Wallis test. If the Epsilon square value is around 0.01, it is interpreted as small; around 0.09, it is interpreted as medium and 0.25 and above as large effect size [42].

The qualitative dataset of the study was transferred to the MAXQDA 2020 Plus qualitative data analysis program and analyzed using the content analysis method. Such software provides researchers with a systematic approach to working with large amounts of qualitative data, making comparisons between categories, and extracting themes from the data [43].

In the analysis of the qualitative data of the study, all authors coded together and continued the analysis until they achieved harmony in the coding and theme creation processes. The interview data were reviewed by two independent researchers before coding. This process was implemented to ensure internal consistency in the interpretation of the codes and to minimize bias. In addition, the constant comparison method was used during coding. In this way, it was aimed to ensure the accuracy of the themes by comparing the similarities and differences between the data. A feedback loop was also provided with the participants through the WhatsApp application during the analysis process. The participants had the opportunity to review the drafts of the interview analysis and contribute with their comments. This feedback process was carried out to support the findings of the study and to increase the accuracy of the researchers' comments. Finally, in the findings section, the findings were supported by direct quotes from the participants' statements (using abbreviations such as F1: 1. Female, M2: 2. Male).

Findings

- Some statistical values are reported without context (e.g., p-values without effect sizes).
- Consider using tables/figures to present key findings more clearly.

Findings on the Analysis of Quantitative Data

Findings Regarding the Global Citizenship Scale and Its Sub-Dimensions

Table 1. Descriptive Statistics on the Global Citizenship Scale

Factors	Min.	Max.	Mean	Sd	Skewness	Kurtosis	Shapiro-Wilk
Normative Environment	4,00	28,00	17,82	6,01	-,353	-,364	,000
Global Awareness	4,00	28,00	18,95	5,90	-,468	-,273	,000
Global Citizenship Identification	2,00	14,00	9,07	3,35	-,292	-,605	,000
Intergroup Empathy	2,00	14,00	9,80	3,29	-,582	-,416	,000
Valuing Diversity	2,00	14,00	10,37	3,26	-,801	-,048	,000
Social Justice	2,00	14,00	10,88	3,37	-1,081	,302	,000
Environmental Sustainability	2,00	14,00	10,77	3,30	-1,036	,326	,000
Intergroup Helping	2,00	14,00	10,51	3,43	-,834	-,195	,000
Responsibility to Act	2,00	14,00	9,98	3,25	-,749	-,090	,000
Global Citizenship Total Score	22,00	154,00	108,20	28,23	-,824	,542	,000

When Table 1 is examined, it is observed that individuals for the normative environment sub-dimension of the global citizenship scale are $min = 4{,}00; maks = 28{,}00;\ \bar{X} = 17{,}82 \pm 6{,}01$. For the global awareness sub-dimension, it was observed that individuals had $min = 4{,}00; maks = 28{,}00;\ \bar{X} = 18{,}95 \pm 5{,}90$. For the global citizenship identification sub-dimension, it was observed that individuals had $min = 2{,}00; maks = 14{,}00;\ \bar{X} = 9{,}07 \pm 3{,}35$. For the intergroup empathy sub-dimension, it was observed that individuals had $min = 2{,}00; maks = 14{,}00;\ \bar{X} = 9{,}80 \pm 3{,}29$. For the valuing diversity sub-dimension, it was observed that individuals had $min = 2{,}00; maks = 14{,}00;\ \bar{X} = 10{,}37 \pm 3{,}26$. For the Social Justice sub-dimension, it was observed that individuals had $min = 2{,}00; maks = 14{,}00;\ \bar{X} =$

$10{,}88 \pm 3{,}37$. For the environmental sustainability sub-dimension, it was observed that individuals had $min = 2{,}00; maks = 14{,}00;\ \bar{X} = 10{,}77 \pm 3{,}30$. For the intergroup helping sub-dimension, it was observed that individuals had min=2.00; max=14.00; X̅=10.51±3.43. For the responsibility to act sub-dimension, it was observed that individuals had $min = 2{,}00; maks = 14{,}00;\ \bar{X} = 9{,}98 \pm 3{,}25$. For the global citizenship total score, it was observed that individuals had $min = 22{,}00; maks = 154{,}00;\ \bar{X} = 108{,}20 \pm 28{,}23$.

Table 2. Scoring System Used in the Global Citizenship Scale

	**Very Low**	**Low**	**Medium**	**High**	**Very High**
Items	1,00-2,49	2,50-3,99	4,00-5,49	5,50-6,49	6,50-7,00

When Table 2 is examined, cut-off scores were determined on an item basis in order to determine the levels of the Global Citizenship Scale. When determining the cut-off scores, five categories were determined to determine the global citizenship levels of the group according to the 7-point Likert scale. Then, the average scores for the sub-dimensions and the main scale dimension were divided by the number of items to determine the levels. Accordingly,

1.00-2.49 is at a very low level

2.50-3.99 is at a low level

4.00-5.49 is at a medium level

5.50-6.49 is at a high level

6.50-7.00 is at a very high level

When the mean scores of the normative environment dimension of the global citizenship scale are examined, it is seen that the group is a citizen of the normative environment at a moderate level $(\bar{X} = 4{,}45)$. When the average of the global awareness dimension scores is examined, it is seen that the group has a medium level of global awareness $(\bar{X} = 4{,}73)$. When the global citizenship identification dimension average is examined, it is seen that the group has

a medium level of global citizenship identification ($\bar{X} = 4{,}53$). When the intergroup empathy dimension average was examined, it was seen that the group had a medium level of intergroup empathy ($\bar{X} = 4{,}90$). When the mean of the valuation diversity dimension was examined, it was seen that the group had a medium level of valuation diversity ($\bar{X} = 5{,}18$). When the average of the social justice dimension was examined, it was seen that the group had a moderate level of social justice ($\bar{X} = 5{,}44$). When the environmental sustainability dimension average was examined, it was seen that the group had a medium level of environmental sustainability ($\bar{X} = 5{,}38$). When the mean of the intergroup helping dimension was examined, it was seen that the group had a medium level of intergroup helping ($\bar{X} = 5{,}25$). When the mean of the responsibility to act dimension was examined, it was seen that the group had a medium level of responsibility to act ($\bar{X} = 4{,}99$). When the average of the global citizenship scale scores was examined, it was observed that the group was a moderate level ($\bar{X} = 4{,}91$) of global citizens.

Findings Regarding the Regional Variable

Table 3. Kruskal Wallis H Test on Country Variable

Variable	Geographical Region	N	X_{mean}	S_x	X^2	sd	p	Difference	ε^2
Normative Environment	Asia	60	18.52	6.16	7.13	4	.129		
	Europe	154	18.79	5.40					
	Russia/Eurasia	118	17.20	6.04					
	Sub-Saharan Africa	103	17.77	6.89					
	Middle East/North Africa	199	17.27	5.88					
Global Awareness	Asia	60	18.58	6.03	18.89	4	.001	2>3	.030
	Europe	154	20.52	5.08				2>5	
	Russia/Eurasia	118	18.47	6.08					
	Sub-Saharan Africa	103	19.28	6.57					
	Middle East/North Africa	199	17.98	5.78					
Global Citizenship Identification	Asia	60	9.32	3.51	1.24	4	.871		
	Europe	154	9.01	3.20					
	Russia/Eurasia	118	9.10	3.32					
	Sub-Saharan Africa	103	9.28	3.48					
	Middle East/North Africa	199	8.93	3.39					
Intergroup Empathy	Asia	60	9.68	3.20	3.24	4	.519		
	Europe	154	10.23	3.03					
	Russia/Eurasia	118	9.56	3.49					
	Sub-Saharan Africa	103	9.60	3.45					

	Middle East/North Africa	199	9.76	3.33					
Valuing Diversity	Asia	60	10.85	3.31	2.98	4	.561		
	Europe	154	10.45	2.81					
	Russia/Eurasia	118	10.07	3.39					
	Sub-Saharan Africa	103	10.45	3.30					
	Middle East/North Africa	199	10.30	3.48					
Social Justice	Asia	60	10.83	3.36	3.22	4	.522		
	Europe	154	11.36	2.84					
	Russia/Eurasia	118	10.44	3.55					
	Sub-Saharan Africa	103	10.77	3.62					
	Middle East/North Africa	199	10.87	3.53					
Environmental Sustainability	Asia	60	10.73	3.17	10.65	4	.031	2>3	.017
	Europe	154	11.44	2.74					
	Russia/Eurasia	118	10.10	3.43					
	Sub-Saharan Africa	103	10.96	3.32					
	Middle East/North Africa	199	10.58	3.58					
Intergroup Helping	Asia	60	10.58	3.42	5.94	4	.204		
	Europe	154	10.55	2.99					
	Russia/Eurasia	118	10.13	3.23					
	Sub-Saharan Africa	103	10.80	3.54					
	Middle East/North Africa	199	10.56	3.81					
Responsibility to Act	Asia	60	10.27	2.97	8.14	4	.086		
	Europe	154	10.05	3.10					
	Russia/Eurasia	118	9.48	3.02					
	Sub-Saharan Africa	103	10.43	3.45					
	Middle East/North Africa	199	9.91	3.47					
Global Citizenship Total Score	Asia	60	109.37	29.46	5.98	4	.201		
	Europe	154	112.40	24.09					
	Russia/Eurasia	118	104.56	27.59					
	Sub-Saharan Africa	103	109.33	30.78					
	Middle East/North Africa	199	106.18	29.60					

When table 3 is examined, it is seen that there is no statistically significant difference between the normative environment, global awareness, global citizenship identification, intergroup empathy, valuing diversity, social justice, environmental sustainability, intergroup helping, responsibility to act and global citizenship total score means according to the country variable; X^2=7.13,p=.129; X^2=1.24, p=.871; X^2=3.24, p=.519; X^2=2.98, p=.561; X^2=3.22, p=.522; X^2=5.94, p=.204; X^2=8.14, p=.086; X^2=5.98, p=.201. It was observed that there was a statistically significant difference between the global awareness and environmental sustainability score averages according to the country variable; X^2= 18.89, p=.001;X^2=10.65, p=.031. Dwass-Steel-Critchlow-Fligner pairwise comparison test was performed to determine

between which groups the significant difference was. As a result of the pairwise comparisons, it was seen that the mean score of Europe for the global awareness sub-dimension was significantly higher than the mean score of Russia/Eurasia and the Middle East/North Africa; and the mean score of Europe for the environmental sustainability sub-dimension was significantly higher than the mean score of Russia/Eurasia. When the effect size of the difference in question was calculated, it could be said that it was small.

When the global citizenship scale total score averages are examined according to geographical regions, the European group ($X_{mean} = 112{,}40$) is the highest, the Asian group ($X_{mean} = 109{,}37$) is second, the Sub-Saharan African group ($X_{mean} = 109{,}33$) is third, the Middle East/North African group ($X_{mean} = 106{,}18$) is fourth and the Russian/Eurasian group ($X_{mean} = 104{,}56$) is last.

Findings Regarding the Social Media Variable

The frequencies of the social media tools used by the participants are given in Table 4.

Table 4. Frequency Values of Social Media Tools Used

**Social Media Tool**	**N**	**%**
Discord	80	12.62
Facebook	398	62.78
Instagram	510	80.44
LinkedIn	173	27.29
Pinterest	150	23.66
Reddit	43	6.78
TikTok	305	48.11
Twitter (now X)	271	42.74

It was observed that the most frequently used social media tools by the participants were Instagram (510) and Facebook (398); the least frequently used social media tools were Reddit

(43) and Discord (80). The correlation coefficient for the social media score and scale score was calculated and is given in Table 5.

Table 5. Spearman Brown Test for Social Media Score

N=634	Sosyal Medya Skoru	p değeri
Normative Environment	.024	.552
Global Awareness	.072	.072
Global Citizenship Identification	.111	.005
Intergroup Empathy	.066	.096
Valuing Diversity	.004	.924
Social Justice	.013	.750
Environmental Sustainability	-.014	.717
Intergroup Helping	.039	.327
Responsibility to Act	.049	.218
Global Citizenship Total Score	.059	.140

It was observed that there was no statistically significant relationship between the social media score and normative environment, global awareness, intergroup empathy, valuing diversity, social justice, environmental sustainability, intergroup helping, responsibility to act and global citizenship total score, $p>0.05$; and there was a statistically significant positive and small-level relationship with global citizenship identification, $p<0.05$.

Findings Regarding the Gender Variable

Table 6. Findings Regarding the Mann Whitney U Test for the Gender Variable

Variable	Gender	N	X_{mean}	Median	S_x	U	p	Binary Correlation
Normative Environment	Male	311	17.38	18.00	6.14	46665	.122	
	Female	323	18.26	19.00	5.88			
Global Awareness	Male	311	19.04	20.00	6.14	48593	.478	
	Female	323	18.88	19.00	5.67			
Global Citizenship Identification	Male	311	8.89	9.00	3.50	47902	.310	
	Female	323	9.26	9.00	3.20			
Intergroup Empathy	Male	311	10.04	10.00	3.28	45729	.049	.090
	Female	323	9.57	10.00	3.30			
Valuing Diversity	Male	311	10.23	11.00	3.50	49153	.638	
	Female	323	10.51	11.00	3.02			

Social Justice	Male	311	10.72	12.00	3.53	48067	.340	
	Female	323	11.06	12.00	3.22			
Environmental Sustainability	Male	311	10.77	12.00	3.50	48368	.413	
	Female	323	10.79	12.00	3.11			
Intergroup Helping	Male	311	10.49	12.00	3.63	49111	.623	
	Female	323	10.54	11.00	3.24			
Responsibility to Act	Male	311	9.80	10.00	3.43	48099	.353	
	Female	323	10.15	11.00	3.08			
Global Citizenship Total Score	Male	311	107.35	113.00	30.34	50186	.986	
	Female	323	109.02	113.00	26.06			

When Table 6 is examined, it is seen that there is no statistically significant difference between the normative environment, global awareness, global citizenship identification, valuing diversity, social justice, environmental sustainability, intergroup helping, responsibility to act, global citizenship total score averages according to the gender variable; U=46665, p=.122; U=48593, p=.478; U=47902, p=.310; U=49153, p=.638; U=48067, p=.340; U= 48368, p=.413; U= 49111, p=.623; U=48099, p=.353; U=50186, p=.986.

It was observed that there was a statistically significant difference between the intergroup empathy scores according to the gender variable, U= 45729, p=.049. The intergroup empathy mean score of men is significantly higher than the mean score of women. When the effect size of the difference in question is calculated, it can be said that it is small.

Findings Regarding the Global Event Participation Variable

Table 7. Findings Regarding the Mann Whitney U Test for the Global Event Participation Variable

Variable	Participation in Global Event	N	X_{mean}	Median	S_x	U	p	Binary Correlation
Normative Environment	No	503	17.44	17.00	5.98	26064	.001	.209
	Yes	131	19.3	20.0	5.95			
Global Awareness	No	503	18.41	18.00	5.83	23684	.001	.281
	Yes	131	21.1	22.0	5.71			
Global Citizenship Identification	No	503	8.75	9.00	3.34	23654	.001	.282
	Yes	131	10.4	10.0	3.07			
Intergroup Empathy	No	503	9.58	10.00	3.31	26111	.001	.207
	Yes	131	10.7	11.0	3.13			
Valuing Diversity	No	503	10.10	10.00	3.21	23582	.001	.284
	Yes	131	11.4	13.0	3.25			

Social Justice	No	503	10.70	12.00	3.42	27335	.002	.170
	Yes	131	11.6	13.0	3.12			
Environmental Sustainability	No	503	10.59	11.00	3.37	27793	.005	.156
	Yes	131	11.5	12.0	2.97			
Intergroup Helping	No	503	10.27	11.00	3.44	25500	.001	.226
	Yes	131	11.5	13.0	3.23			
Responsibility to Act	No	503	9.75	10.00	3.23	25339	.001	.231
	Yes	131	10.9	12.0	3.22			
Global Citizenship Total Score	No	503	105.59	110.00	27.83	22854	.001	.306
	Yes	131	118.2	125.0	27.61			

When Table 7 is examined, it is seen that there is a statistically significant difference between the averages of normative environment, global awareness, global citizenship identification, intergroup empathy, valuing diversity, social justice, environmental sustainability, intergroup helping, responsibility to act and global citizenship total score according to the variable of participation in global events; U= 26064, p=.001; U=23684, p=.001; U=23654 p=.001; U=26111 p=.001; U=23582 p=.001; U=27335 p=.001; U=27793 p=.001; U=25500 p=.001; U=25339 p=.001; U=22854 p=.001. The sub-dimension and total score averages of the participants who participated in a global event are significantly higher than the score averages of the participants who did not participate. When the effect size for the difference in question is calculated, it can be said that it is medium for the total score and small for the sub-dimensions.

Findings Regarding the Variable of Being a Member of a Global Association

Table 8. Findings Regarding the Mann Whitney U Test for the Variable of Being a Member of a Global Association

**Variable**	**Membership Status**	**N**	X_{mean}	**Median**	S_x	**U**	**p**	**Biserial Correlation**
Normative Environment	No	532	17.59	18.00	5.94	22580	.007	.168
	Yes	102	19.08	20.0	6.32			
Global Awareness	No	532	18.72	19.00	5.66	21912	.002	.192
	Yes	102	20.20	22.0	6.91			
Global Citizenship Identification	No	532	8.92	9.00	3.29	21917	.002	.192
	Yes	102	9.89	10.0	3.58			
Intergroup Empathy	No	532	9.73	10.00	3.26	24204	.082	
	Yes	102	10.20	11.0	3.49			
Valuing Diversity	No	532	10.26	11.00	3.20	22499	.006	.171

	Yes	102	10.97	12.0	3.51			
Social Justice	No	532	10.83	12.00	3.32	23920	.053	
	Yes	102	11.19	13.0	3.69			
Environmental Sustainability	No	532	10.75	11.00	3.24	25312	.275	
	Yes	102	10.91	12.0	3.63			
Intergroup Helping	No	532	10.39	11.00	3.36	21711	.001	.200
	Yes	102	11.20	13.0	3.72			
Responsibility to Act	No	532	9.90	10.00	3.15	22875	.011	.157
	Yes	102	10.42	12.0	3.78			
Global Citizenship Total Score	No	532	107.08	112.00	26.75	20938	.001	.228
	Yes	102	114.05	128.0	34.52			

When Table 8 is examined, it is seen that there is no statistically significant difference between the mean scores of intergroup empathy, social justice and environmental sustainability according to the variable of being a member of a global association; U=24204, p=.082; U=23920, p=.053; U=25312, p=.275. According to the variable of being a member of a global association, there is a statistically significant difference between the averages of normative environment, global awareness, global citizenship identification, valuing diversity, intergroup helping, responsibility to act and global citizenship total score.; U= 22580, p=.007; U=21912, p=.002; U=21917, p=.002; U=22499, p=.006; U=21711, p=.001; U=22875, p=.011; U=20938, p=.001. It was observed that the mean sub-dimension scores of the participants who were members of the association were significantly higher than those who were not members of the association. When the effect size for this difference was calculated, it could be said that it was small.

Findings Regarding Age Variable

Table 9. Kruskal Wallis H Test for Age Variable

Variable	Age	N	X_{ort}	S_x	X^2	sd	p	Difference	ε^2
Normative Environment	18-20	83	17.04	5.49	4.64	3	.200		
	21-23	228	17.95	5.79					
	24-26	125	17.36	6.28					
	27+	198	18.31	6.31					
Global Awareness	18-20	83	18.49	5.66	3.85	3	.278		
	21-23	228	19.05	5.43					
	24-26	125	18.28	6.20					
	27+	198	19.48	6.30					
	18-20	83	8.67	2.99	6.40	3	.094		
	21-23	228	8.86	3.31					

Global Citizenship Identification	24-26	125	9.09	3.51					
	27+	198	9.49	3.42					
Intergroup Empathy	18-20	83	9.61	2.97	24.41	3	.001	1<4	.039
	21-23	228	9.19	3.35				2<4	
	24-26	125	9.62	3.62					
	27+	198	10.70	2.96					
Valuing Diversity	18-20	83	10.39	2.75	16.61	3	.001	2<4	.026
	21-23	228	9.94	3.35				3<4	
	24-26	125	10.04	3.47					
	27+	198	11.07	3.13					
Social Justice	18-20	83	10.51	2.94	19.93	3	.001	1<4	.031
	21-23	228	10.75	3.55				2<4	
	24-26	125	10.22	3.66				3<4	
	27+	198	11.64	3.04					
Environmental Sustainability	18-20	83	10.33	2.84	16.30	3	.001	1<4	.026
	21-23	228	10.76	3.44				3<4	
	24-26	125	10.25	3.41					
	27+	198	11.32	3.20					
Intergroup Helping	18-20	83	10.02	2.94	28.93	3	.001	1<4	.046
	21-23	228	10.15	3.59				2<4	
	24-26	125	9.97	3.58				3<4	
	27+	198	11.48	3.16					
Responsibility to Act	18-20	83	9.55	2.76	20.45	3	.001	1<4	.032
	21-23	228	9.77	3.22				2<4	
	24-26	125	9.50	3.48				3<4	
	27+	198	10.70	3.25					
Global Citizenship Total Score	18-20	83	104.61	23.56	17.52	3	.001	1<4	.028
	21-23	228	106.41	27.42				2<4	
	24-26	125	104.34	31.95				3<4	
	27+	198	114.20	27.68					

When Table 9 is examined, it is seen that there is no statistically significant difference between the normative environment, global awareness and global citizenship identification mean scores according to the age variable.; X^2 =4.64, p=.200;X^2 =3.85, p=.278; X^2= 6.40, p=.094. It was observed that there was a statistically significant difference between the averages of intergroup empathy, valuing diversity, social justice, environmental sustainability, intergroup helping, responsibility to act and global citizenship total score according to the age variable; X^2= 24.41, p=.001;X^2= 16.61, p=.001;X^2= 19.93, p=.001;X^2= 16.30, p=.001;X^2= 28.93, p=.001;X^2= 20.45, p=.001;X^2= 17.52, p=.001. The Dwass-Steel-Critchlow-Fligner pairwise comparison test was conducted to determine between which groups the significant difference occurred. As a result of the pairwise comparisons, it was observed that the sub-dimension scores of participants over the age of 27 were significantly higher than those of other

age groups. When the effect size for the difference in question was calculated, it could be said that it was small.

Findings on the Analysis of Qualitative Data

Findings Regarding Students' Definitions of Global Citizenship

In the interview conducted with the students in the study group, they were asked how they defined global citizenship. The students' responses are shown in Figure 1:

Figure 1. Maxmap of participants' definitions of global citizenship

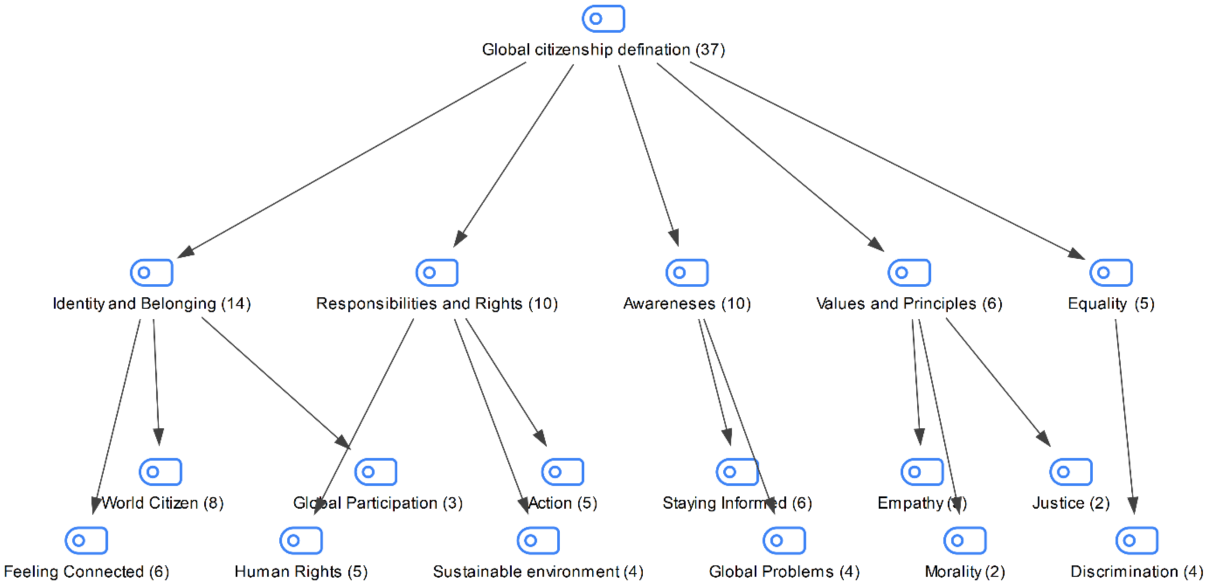

When the responses of the students regarding their definitions of global citizenship are examined, it is seen that the majority of the students explain the concept of global citizenship mostly through "identity and belonging, responsibilities and rights". It can be said that the students consider the concept of global citizenship in a multidimensional framework and see global citizenship as a concept that starts with the identity and belonging of individuals, is shaped by awareness, responsibility and values, and is completed with themes such as equality and justice.

Some of the students' responses are as follows:

“Someone who understands the world and feels part of it, no matter the nation and race. A person who feels like they belong with everyone else in the world socially, politically, economically etc.” (F8)

“Global citizenship is the recognition that we are all part of a shared humanity, with a responsibility to respect diversity, act ethically, and contribute to the global community’s well-being.” (M12)

Findings on Whether Students See Themselves as Global Citizens

In the interview conducted with the students in the study group, they were asked whether they saw themselves as global citizens. The students' responses are shown in Figure 2:

Figure 2. Maxmap of whether the participants see themselves as global citizens

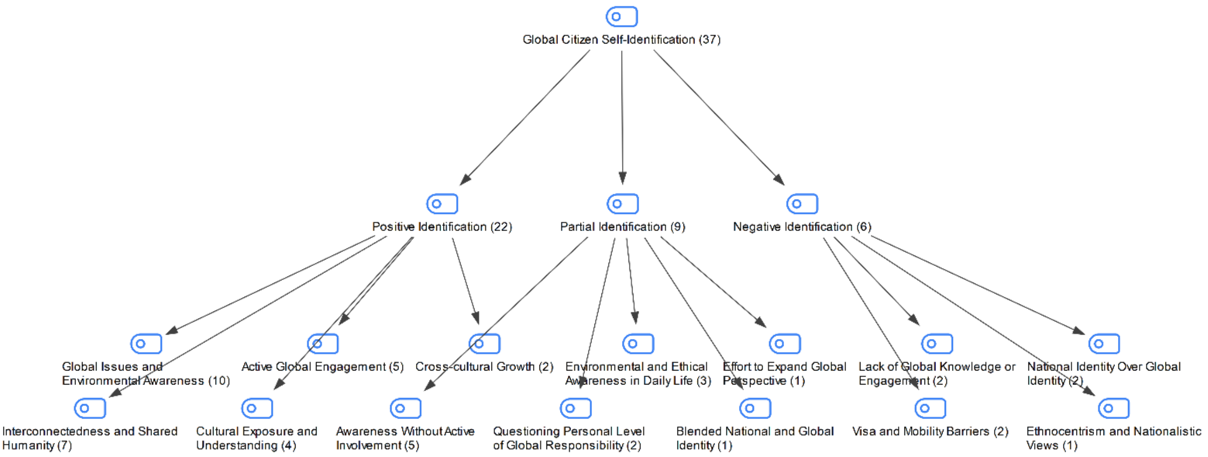

When the students' answers to the question of whether they see themselves as global citizens are examined, it is seen that the majority of the students define themselves as global citizens. The rate of those who gave a positive opinion (f 22) shows that the majority of the students are open to the idea of global citizenship. Although this shows that the students are inclined to adopt the global citizenship identity, it can be said that some of them lack active participation. The rates of those who gave a partial answer (f 9) and a negative answer (f 6) draw attention to the

lack of awareness, knowledge or conflicts between national identity and global identity. The group that gave a partial answer may have given this answer due to a lack of national identity, ethical responsibility or awareness, although they are open to the concept of global citizenship. It can be said that the group that gave a negative opinion cannot establish a connection with global citizenship due to lack of knowledge, national identity preference or ethnocentric views.

Some of the students' responses are as follows:

> "Yes, because I actively engage in issues that affect the world beyond my immediate environment, seek to understand diverse perspectives, and aim to contribute positively to global challenges." (M9)
>
> "I am aware of world's problems, like pollution, global warming but I haven't done anything about that, didn't participate any activity." (F17)
>
> "No, I don't. Because I have a strong identity of citizen of my country." (F2).

Findings on the Skills, Knowledge and Attitudes that Global Citizens Should Have, According to Students

In the interview conducted with the students in the study group, the skills, knowledge and attitudes that global citizens should have were asked. The students' responses are shown in Figure 3:

Figure 3. Maxmap of the skills, knowledge and attitudes that global citizens should have according to the participants

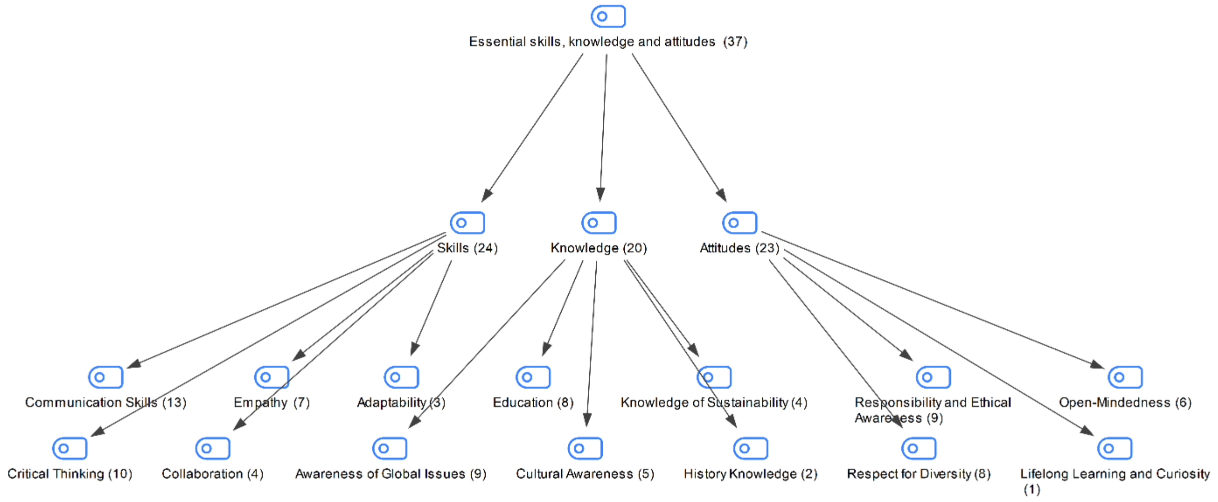

When the answers given by the students regarding the skills, knowledge and attitudes required for global citizenship are examined, they stated that a global citizen should generally have communication, critical thinking and empathy skills, global awareness, sustainability knowledge, responsibility and ethical awareness, and respect for diversity. The emphasis on skills such as communication, empathy and critical thinking may be an indication that students care about the practical and social dimensions of global citizenship. We can say that the prominence of issues such as global issues and sustainability shows that students are conscious of world problems. The prominence of ethical awareness and respect for diversity may be an expression of students' sensitivity to the concepts of justice and equality.

Some of the students' responses are as follows:

> "Acceptance of different cultures, understanding issues on global level and self-identification of one a global citizen (I think it's a must to understand we are all the same people in the world, just with differences that should not matter)." (M10)

> "For me global citizen should have critical thinking, empathy, cultural awareness, adaptability, and a commitment to social justice are essential for global citizenship, as they enable individuals to engage constructively in global issues." (M17)

"Someone who is tolerant, open-minded and respectful of the social community's way of life should be someone who thinks that people are of the world and belong to the world, and accepts the earth as a whole without borders and cares about sustainability in every action." (F4)

Findings on Students' Responses to the Responsibilities of Global Citizens to Promote Prosperity and Justice in the World

In the interview conducted with the students in the study group, they were asked about the responsibilities of global citizens to promote prosperity and justice in the world. The students' responses are shown in Figure 4:

Figure 4. Maxmap of participants' responses regarding the responsibilities of global citizens to promote prosperity and justice in the world.

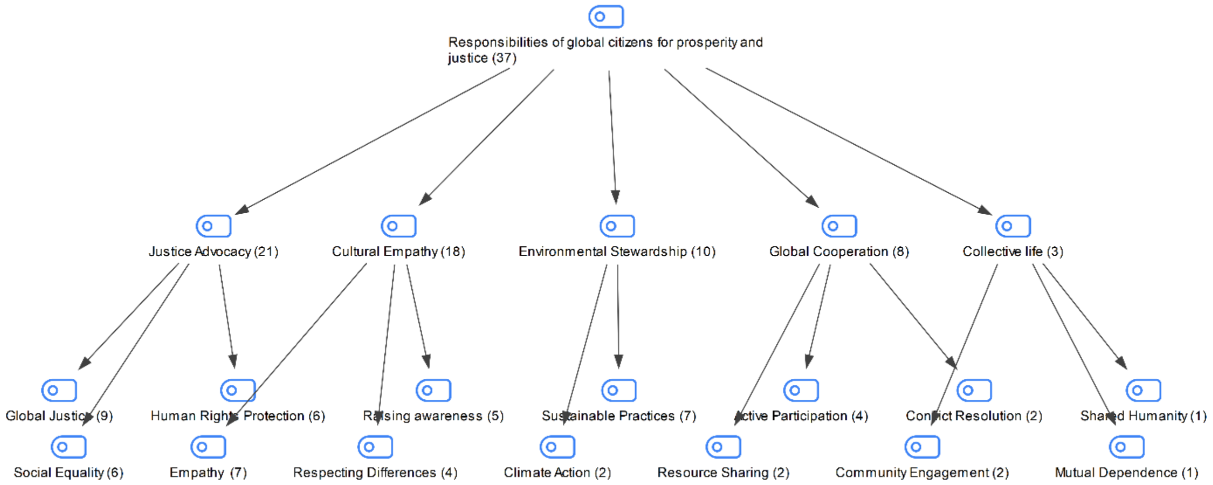

When the students' responses regarding the responsibilities of global citizens to promote prosperity and justice in the world are examined, the students stated that global citizens should generally be advocates of justice, have cultural empathy and be sensitive to the environment in order to promote prosperity and justice in the world. The students addressed the responsibilities of global citizens from various perspectives and emphasized the issues of advocacy of justice, cultural empathy and environmental responsibility the most. We can say that these statements

show that the students have a broader perspective on global problems beyond individual steps and that they see cooperation and solidarity as important.

Some of the students' responses are as follows:

> "Understanding of global issues and participating in fixing those (actively and passively). Empathy for everyone no matter their culture, religion or race." (M11)

> "Individuals should strive to promote social justice, protect the environment, and support global cooperation to enhance prosperity and fairness worldwide." (M3)

Findings Regarding Students' Responses to the Question "The World's Biggest Problems"

In the interview conducted with the students in the study group, the biggest problems in the world were asked. The answers of the students are shown in Figure 5:

Figure 5. Maxmap of the participants' responses to the question of the biggest problems in the world

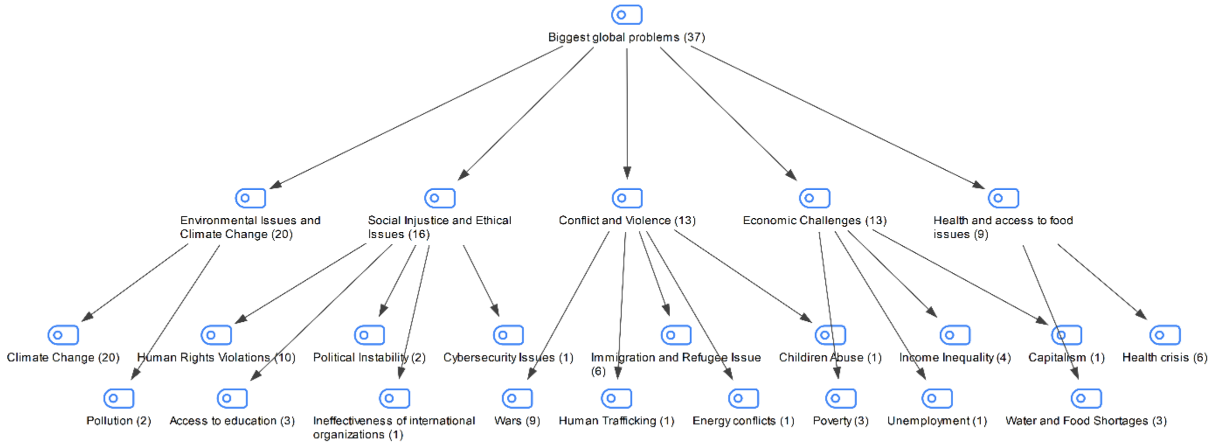

When the answers given by the students about the biggest problems in the world are examined, the answers given by the students about the biggest problems in the world are gathered under

the themes of environmental problems and climate change, social injustice and ethical problems, conflict and violence, health and economic difficulties. These themes show that the students address the biggest problems in the world in a very comprehensive and different perspective. While environmental problems and climate change come first, basic issues such as social injustice and economic difficulties also come to the fore. It is possible to say that the emphasis on conflict and violence, health and food access issues is sensitive to problems on both an individual and global scale.

Some of the students' responses are as follows:

> "The biggest global problems include climate change, income inequality, and political instability." (F12)
>
> "Climate change, health crisis, inequality, conflict and violence." (F13)

Findings Regarding Students' Question on What Sustainability Means to Them

In the interview with the students in the study group, they were asked what sustainability meant to them. The students' answers are shown in Figure 6:

Figure 6. Maxmap of participants' views on what sustainability means to them.

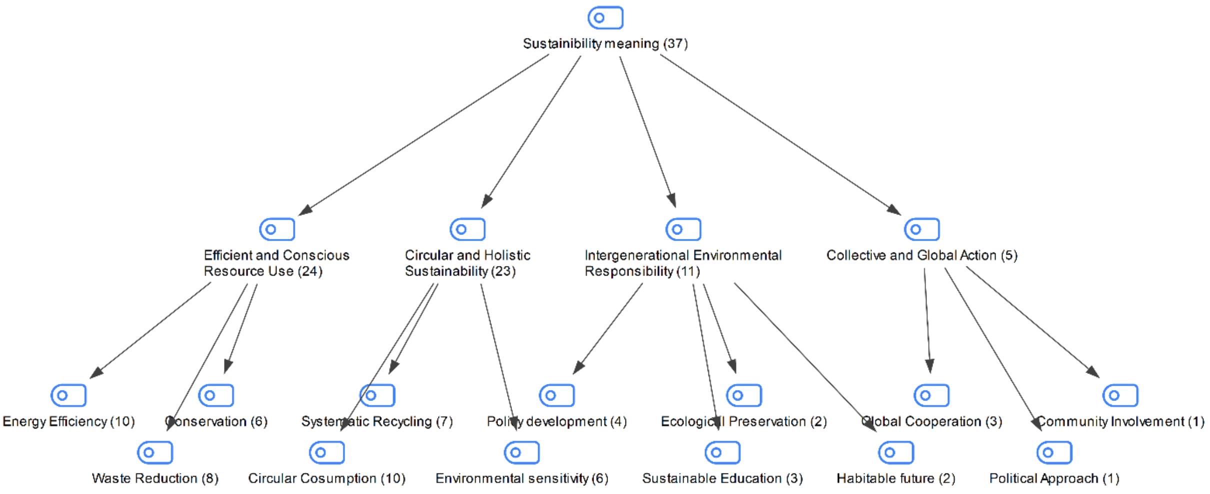

When the students' answers to the question about what sustainability means to them are examined, it is seen that sustainability is expressed as a multidimensional concept that should be addressed both individually and globally. Students mostly associated sustainability with environmental actions such as efficient use of resources, waste reduction and recycling. In addition, social dimensions such as policy development and sustainable education also have an important place in students' understanding of sustainability. Students also see sustainability as a more comprehensive concept with elements such as protecting future generations and global cooperation. It is possible to explain these answers of the students as having a collective responsibility for solving environmental and social problems.

Some of the students' responses are as follows:

> "For me sustainability is a balanced approach to environmental health, social equity and economic viability." (F14)

> "Sustainability means ensuring that we take care of the environment enough to make sure that future generations can live in a safe world." (M1)

Findings on Students' Actions in Their Daily Lives for a Sustainable World

In the interview conducted with the students in the study group, they were asked about the actions they take in their daily lives for a sustainable world. The students' answers are shown in Figure 7:

Figure 7. Maxmap of the actions taken by the participants in their daily lives for a sustainable world.

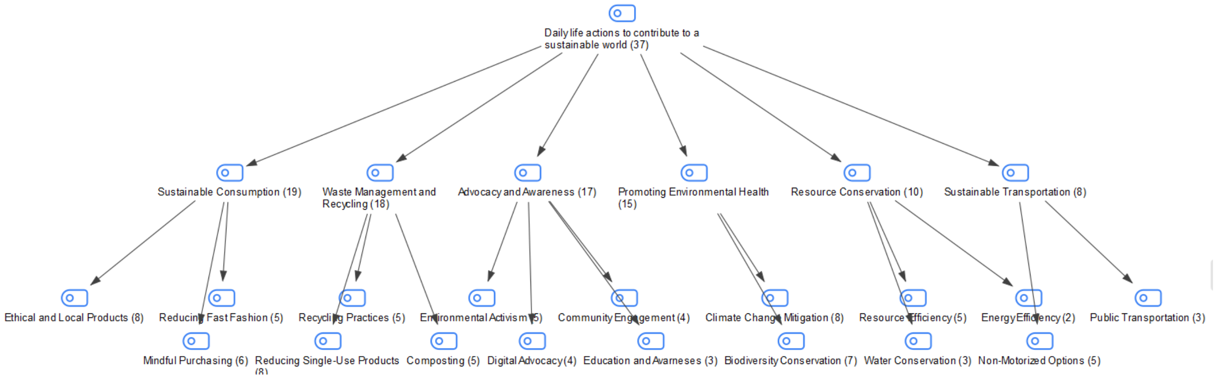

When the answers given by the students to the question about the actions they take in their daily lives for a sustainable world are examined, we can say that the students generally carry out actions such as sustainable consumption habits, waste management and recycling, and conservation of resources in their daily lives for a sustainable world. In line with the answers received, it is possible to say that the students have a global citizenship awareness and that they put this awareness into practice in their daily lives. In addition, the fact that they act by considering both individual and social impacts show that they adopt an approach compatible with sustainable development goals. In addition, the actions of the students are very important in the long term in terms of both starting to transform their individual habits and aiming to create awareness for society.

Some of the students' responses are as follows:

> "I do not use water and electricity unnecessarily. I compost food waste in the garden. I throw plastic bottle paper waste into recycling bins. By following world developments on social media and newspapers, I tell and offer people who are around me world should be without war and more clean." (F5)

> "Consuming less and more intentionally, using green energy sources and talking public transportation." (F14)

Findings Regarding Students' Responses to the Impact of Global Citizenship on Sustainability

In the interview conducted with the students in the study group, the impact of global citizenship on sustainability was asked. The students' responses are shown in Figure 8:

Figure 8. Maxmap of participants’ views on the impact of global citizenship on sustainability

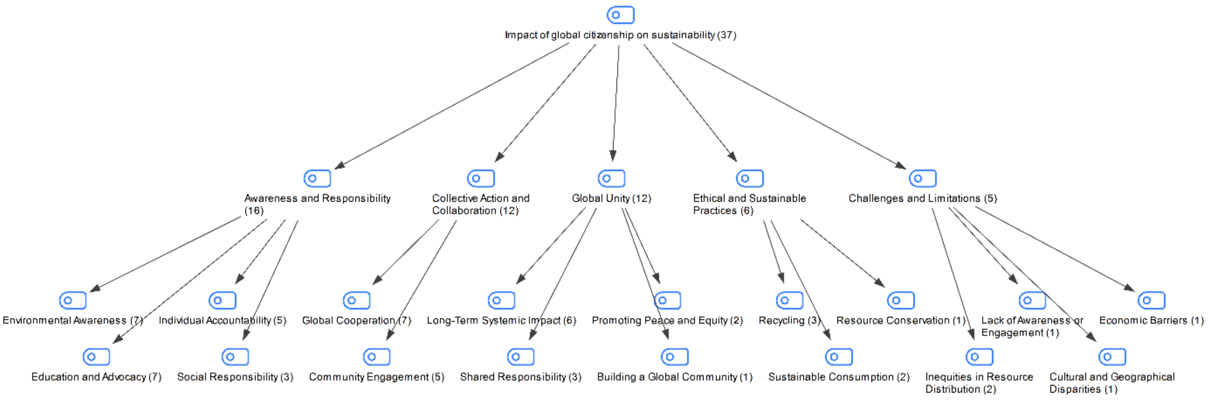

When we look at the students' answers to the question about the impact of global citizenship on sustainability, we can say that the students generally explain the impact of global citizenship on sustainability under the themes of awareness and responsibility, global unity, cooperation and joint action, ethical and sustainable practices, challenges and limitations. The students have addressed the impact of global citizenship on sustainability quite comprehensively with their answers. This shows that they have a conscious and responsible global perspective. In addition, while emphasizing positive aspects such as awareness, collective cooperation, and ethical practices, the students also drew attention to challenges such as resource inequality and economic barriers. In short, the students evaluated the impact of global citizenship on sustainability in a balanced way, both in terms of opportunities and limitations. It is possible to say that this shows that they address global problems from a multidimensional perspective.

Some of the students' responses are as follows:

> “Global citizenship encourages individuals to see beyond borders and work collaboratively to address issues like climate change and inequality, amplifying efforts towards a more sustainable future.” (M14)

> “The impact of global citizenship on sustainability can be assessed by looking at how individual and collective actions contribute to sustainable development goals (SDGs) and positive environmental and social outcomes.” (F3)

Findings on the Decisions and Actions Students Would Make/Take If They Were UN Secretary

In the interview conducted with the students in the study group, they were asked about the decisions and actions they would make/take if they were UN secretary. The students' answers are shown in Figure 9:

Figure 9. Maxmap of participants’ definitions of global citizenship

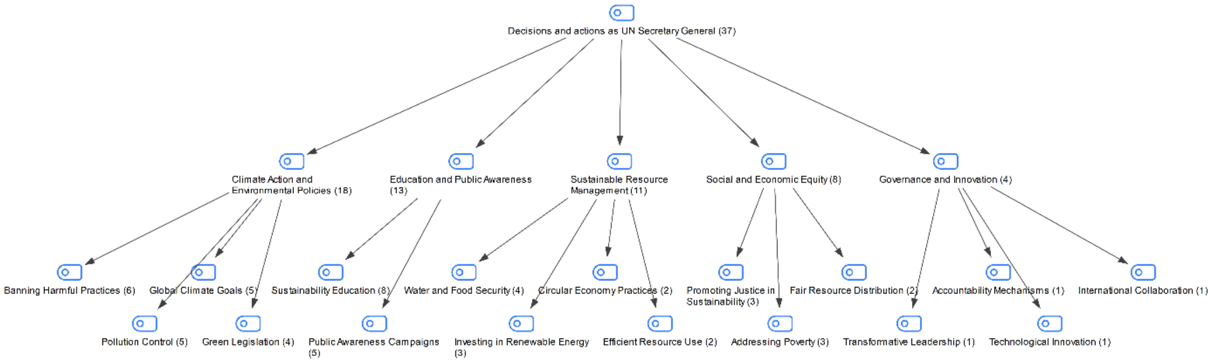

When we look at the students' answers to the question about the decisions and actions they would make/take if they were UN Secretary, we can say that they generally explain the decisions and actions they would make/take under the themes of climate action and environmental policies, education and public awareness, sustainable resource management, social and economic equality, management and innovations. Firstly, it is possible to say that the students' answers exhibit a balanced approach covering both environmental and social dimensions and that they exhibit a strong sustainability awareness and global citizenship understanding. In addition, it is possible to say that the students' prioritization of environmental problems, social equality and education reflects their thinking with a broad perspective and their efforts to produce solutions for an important step such as sustainability.

Some of the students' responses are as follows:

> "Advocate for more plastic bans and try to create sustainable plans for regions that are hit with poverty, in a sense that it's not just one-time solution, but a long term one (giving food will not solve the poverty as we need to think how to create conditions for the people there to live a proper life, where they can themselves create work, food and every condition we have here in more developed regions)." (F16)

> "As UN Secretary General, I would prioritize global collaboration to enforce sustainable policies, such as carbon reduction goals and equitable resource distribution." (M6)

Conclusion and Discussion

When the results of this study, which aims to determine the global citizenship tendencies of international university students, are examined, it is possible to evaluate the international university students in the study group in line with the various dimensions of the Global Citizenship Scale based on the quantitative findings. The general global citizenship tendency of the students in the study group is at a medium level. This shows that the participants are interested in global issues and feel responsible, but this sensitivity is not high. When we look at the literature, it is possible to see studies in which the global citizenship scores of university students are at a medium level [44–50]. Liu et al. [51] revealed in their study that university students in Taiwan generally have a high level of self-awareness regarding global citizenship.

In the study, participants also obtained moderate scores in all dimensions within the scope of the Global Citizenship Scale, but some significant differences are noticed between different dimensions. It is possible to interpret this situation as a significant diversity in the participants' awareness and responsibility levels regarding different global citizenship components. In more detail, it was observed that the participants first obtained higher scores in the dimensions of "social justice, environmental sustainability, value of diversity and intergroup cooperation" compared to the other dimensions, but at a moderate level. This can be said to

indicate that the participants are sensitive about creating a fair society, have awareness of environmental protection and sustainability issues, tend to value different cultures, ideas and individual differences, and care about social solidarity and cooperation. Participants also obtained moderate scores in the dimensions of "ideal environment, global awareness, global citizenship identity, responsibility for action and intergroup empathy" but received lower scores than the other dimensions. This can be said that the participants are sensitive to the environment, but this sensitivity is not at a very high level, they are knowledgeable about global issues but the depth or impact of this knowledge may be limited and they are open to understanding the perspectives of others. In addition, although the participants felt a certain degree of global responsibility in terms of their global citizenship identity, it is possible to say that this identity was not fully strong and that their levels of taking action were slightly lower compared to awareness and empathy in terms of responsibility for action.

When the scores of the participants in terms of variables from the Global Citizenship Scale are examined, the first examination is made according to the regions where the international students come from. When examined regionally, it is seen that the European group has the highest average score on the global citizenship scale. This situation shows that individuals living in this region have a higher level of awareness and responsibility in areas such as global awareness, social justice, environmental sustainability and valuing diversity. The reason for this can be evaluated as the fact that there is generally a stronger democratic infrastructure, higher education level, environmental awareness policies and comprehensive studies on human rights in European countries and participation in international cooperation activities such as the European Union can strengthen the perception of individuals' global citizenship identity.

It is seen that the Asian and Sub-Saharan African groups, whose averages are close to each other, are in second and third place in global citizenship awareness. There are significant

economic, cultural and social differences between different countries in Asia. It can be thought that especially in developed countries, high levels of education and global cooperation policies can increase global citizenship awareness. However, income inequality and environmental problems in the region may be factors that decrease the general average. In the Sub-Saharan African Group, the effort to resist racism and exploitation and the desire to find the place they deserve in the world may have positively reflected in the Global Citizenship Scale scores. However, economic difficulties and limited educational opportunities in this region may have been obstacles to increasing the level of awareness.

The Middle East and North Africa group scores are behind Asia and Sub-Saharan Africa. This may be due to political instability, conflicts, human rights issues and deficiencies in education systems in the region. However, the potential of the young population in these regions, the cultural diversity of individuals and sensitivity to communities do not leave global citizenship awareness at a completely low level. Finally, the lowest score in the Russia/Eurasia group indicates that awareness in global citizenship dimensions is limited. This may be due to limited democratic practices in the region, ongoing conflicts, less emphasis on global cooperation and nationalist policies in some countries.

In literature, although a direct regional study like this one could not be found, it is possible to show similar studies that have been conducted on a country basis and when considered according to regions. For example, in their study with international graduate students from three countries representing three continents, Szelenyi and Rhoads [52] found that Italian students had a more global citizenship perception due to their familiarity with the multi-layered identity understanding of the European Union, while students from Brazil and China focused more on their national identities and the problems of their countries. In their study with university students, Der-Karabetian, Cao and Alfaro [53] found that the perception of national identity was more pronounced in Chinese students, while the perception of global

identity was more prominent in Taiwanese students. In addition, Katzarska-Miller, Reysen, Kamble, and Vithoji [54] revealed that the global citizenship identity of participants from Bulgaria and India was strongly adopted by both groups, with India having stronger values in the principles of "empathy, collectivism, and environmental awareness," while Bulgaria had stronger values in the principles of "social justice and equality." In literature, the study by Gerodimos et al. [48] can be given as an example of a study that is not similar to the findings of this study. The researchers stated that students of African and Asian origin feel globalization more intensely and assume greater responsibility than students of American and Western European origin.

Examining the global citizenship tendencies of international university students according to the regions they come from is important in terms of showing how the social, economic, political and cultural characteristics of the geographical regions in question affect the global citizenship levels of international university students. Although the differences between these regions are evident, the strengths and challenges of each region are revealed. University students are in a group with a medium level of global citizenship awareness but with potential for progress.

The scores they received from the Global Citizenship Scale applied to international university students were also examined by different variables. One of these variables was the effect of social media use on global citizenship tendencies. A positive and significant correlation was found between social media use scores and basic structures such as ideal environment, global awareness, global citizenship identity, intergroup empathy, value of diversity, social justice, environmental sustainability, intergroup cooperation, action responsibility and general global citizenship score. These findings indicate the role of social media in shaping global citizenship tendencies. In the literature it is possible to see examples similar to the findings of this study. For example, Cleofas and Labayo [55] revealed in their

studies that online civic participation and being a student working online positively predicted all areas of global competence. Ekici [56] concluded that social media use affected global citizenship awareness in terms of reaching the whole world, being aware of differences, combating social inequalities, interacting with different cultures, producing solutions to social problems, creating awareness, being sensitive, facilitating information sharing, critical thinking and creating a sense of trust. Ben Ltaifa and Derbali [57] stated that social networks contribute to introducing individuals to global cultures and communicating effectively with these cultures. Lee, Baring, Maria and Reysen [58] revealed in their studies that being a member of more than one social networking site is associated with higher global awareness. In addition, Çakmak, Bulut and Taşkıran [59] stated that as the attitude towards internet use increases, the level of global citizenship also increases. Finally, Maguth and Yamaguchi [60] stated that social networking sites contribute to global citizenship education.

When the gender variable is examined, no statistically significant differences were observed in the dimensions of the Global Citizenship Scale, including global awareness, diversity value, and social justice. However, a significant difference was observed in intergroup empathy scores, where men had slightly higher means than women. However, the effect size was small, and it is possible to say that gender plays a minor role in shaping the dimensions of the scale. When the literature is examined, Kaya [61] similarly revealed that men's global citizenship scores were higher than women, although not statistically significant. There are also studies showing that gender does not have a significant effect [45,62,63]. In contrast to this study, in the study conducted by Bulut [64] and Alsawi [49] with university students, the global citizenship levels of female participants were higher than male participants.

When the age variable is examined, significant differences were observed between age groups, and participants aged 27 and over received higher scores in dimensions such as intergroup empathy, value of diversity, and social justice. It is possible to say that these results

indicate that age and life experience contribute to the development of global citizenship awareness. Sherman's [65] study has findings that support these results. The researcher stated in his study that students' identities as global citizens become stronger as they get older. Apart from this study, there are also studies that show that age does not have a significant effect [62,63]. In addition, Smith et al.'s [66] study reveals that age is negatively correlated with global citizenship.

When the variable of participation in global events was examined in the study, it was seen that those who participated in global events received higher scores in all dimensions of the Global Citizenship Scale compared to those who did not participate. These findings show the effect of experiential learning and participation in global events on understanding global citizenship more effectively. Similarly, Reysen and Hackett [67] stated in their study that participation in activist issues contributes to the identity of global citizenship by making the individual feel connected and informed about the world. Troll [68] also suggests in his study that activist movements can create a stronger global citizenship movement by establishing a connection between traditional civil society organizations and social movements.

In the study, the global association membership variable was associated with higher scores in the areas of ideal environment, global awareness, global citizenship identity, and action responsibility, among other dimensions. Those who were not members of the global association received lower scores. It can be said that these results show the positive effect of civic participation in a global association on better understanding global citizenship. Similarly, Chui and Leung [63] stated that the global citizenship levels of university students were higher among students who participated in non-governmental organizations, international voluntary aid organizations, international educational institutions, and international exchange programs. In addition, Albanesi et al. [69] revealed in their study that members of voluntary organizations

were an important context for the development of global citizenship in young generations compared to non-members.

If the quantitative findings of the study are to be evaluated in general, it is possible to say that international university students generally have a medium level of global citizenship awareness. In the study, it was observed that variables such as social media use, age, regional differences, participation in global activities and membership in global organizations significantly affect global citizenship tendencies. Social media stands out as an important tool in shaping individuals' global citizenship qualities by providing a positive effect especially in areas such as global awareness, social justice and environmental sustainability. It was observed that the age factor was decisive and that older students had higher levels of awareness and responsibility. In addition, regional differences also stood out as an important factor affecting global citizenship tendencies. It was evaluated that students in Europe had a higher level of global citizenship awareness and that this could be related to factors such as democratic infrastructure, education levels and participation in international cooperation structures. On the other hand, students in Asia and Sub-Saharan Africa regions exhibited a medium level of awareness, while students in the Middle East, North Africa and Russia/Eurasia regions were found to have lower levels. However, participation in global activities and membership in global organizations have a strong effect on increasing individuals' global citizenship qualities. These findings show that participation in communities and experiential learning are factors that strengthen global citizenship tendencies. In addition, although gender has a small effect on global citizenship tendencies, no significant difference was found in general, except for men scoring slightly higher in the intergroup empathy dimension.

When we look at the qualitative findings of the research, we can first consider the participants' explanations of the concept of global citizenship. When the qualitative findings regarding the concept of global citizenship are examined, it is seen that the participants shape

this concept with the values of identity, belonging, awareness, responsibility, equality and justice. This shows that the participants understand the concept of global citizenship and evaluate it from a multidimensional perspective. In particular, the prominence of themes such as justice, equality, awareness and values show that the participants evaluate the concept of global citizenship not only at the individual level but also in a social and global sense. It is possible to evaluate these findings as students tend to develop a comprehensive understanding of global citizenship. In literature it is possible to see similar studies. For example, Aydın and Andrews [70] mentioned in their study that for a global citizen, the participants should be people who approach social justice empathetically, are active advocates for justice, unity and equality, are aware of their place in the global society, condemn oppression and human rights violations and can think critically about social and political issues from a global perspective. Aydın et al. [71] revealed in their study that students defined global citizenship as someone who understands different cultures, respects them, and appreciates diversity. Altıkulaç and Yontar [72] stated in their study that prospective teachers defined global citizenship as someone who sees everyone as equal, respects people from different countries, supports global development, and is an interactive citizen. Kuleta-Hulboj [73] revealed in his study that participants defined global citizenship in the context of global awareness and participation in action with the values of knowledge, equality, diversity, and justice. In addition, Massey [74] also stated in his study that participants defined the characteristics of a global citizen as global awareness, belonging, compassion, and commitment to action.

The qualitative findings of the study also show that the proportion of international university students who define themselves as global citizens is high. This shows that the vast majority of students tend to adopt a global citizenship identity. In literature, we can come across similar studies. For example, the results of the World Values Survey (WVS) [75] showed that 71.3% of participants from 60 countries (n=88,724) thought that "I see myself as a global

citizen." In addition, Liu et al. (2023) stated in their study that university students in Taiwan generally have a high level of self-awareness about global citizenship and see themselves as global citizens. Baker and Fang [76] also found in their study that there were generally positive attitudes towards intercultural citizenship among a group of Chinese international students and that many of them thought that this was an identity they wanted to develop or had already developed.

However, it can be thought that students who responded partially or negatively encountered certain obstacles in the process of adopting this identity. These obstacles are likely to include lack of knowledge and awareness and the contradictions they experience between national and global identities. Similarly, in the literature, it is seen that in the study of Altıkulaç [77], 98% of the prospective teachers defined themselves as “patriots”, while 2% defined themselves as “world citizens”. In their study examining the perceptions of prospective teachers about global citizenship, Günel and Pehlivan [78] revealed that the participants could not be considered global citizens because they thought that they did not have some of the characteristics that a global citizen should have, and that the participants had a negative perception of themselves regarding being a global citizen.

The qualitative findings of the study also included the opinions of international university students regarding the skills, knowledge and attitudes required for global citizenship, and it was observed that the skills, knowledge and attitudes that the participants deemed necessary for global citizenship were expressed quite comprehensively. The expression of elements such as global issues, sustainability knowledge, ethical sensitivity and respect for diversity, as well as communication, empathy and critical thinking skills, shows that the students address the concept of global citizenship more in terms of its social dimensions. In addition, the prominence of issues such as global issues and sustainability indicates that students have developed awareness of world problems. The prominence of ethical awareness and respect

for diversity may be an expression of students' sensitivity to universal values such as justice and equality. It is possible to see studies similar to these results of the study. For example, the international aid organization Oxfam [79], which supports global citizen education, has classified the characteristics that a global citizen should have under three headings as Knowledge and Understanding, Skills, Values and Attitudes. The headings in this classification are similar to the answers of international university students. The answers given by the students are also similar to the headings in Hunter's [80] "Global Competence Model". In addition, it is possible to say that the answers of the participants regarding the skills that should be possessed in raising global citizens are similar to those of Akcay et al. [27], who worked with teacher candidates.

In the study, the students' explanations about the responsibilities of global citizens in the context of promoting welfare and justice in the world are focused on basic concepts such as advocacy of justice, cultural empathy and environmental sensitivity. This finding reveals that the students see global citizenship as an area of responsibility that requires solidarity and cooperation at a global level, beyond individual steps. In addition, the emphasis on justice, empathy and environmental sensitivity shows that the students develop awareness of both social and environmental problems and are conscious of taking active roles in solving these problems. It can be said that these answers of the participants show similar characteristics to the definition of global citizenship by the multinational organizations Oxfam [11] and UNICEF [10].

It was observed that the international university students in the study group focused on a wide range of areas such as environmental problems and climate change, social injustice, conflict and violence, health and economic difficulties when addressing global issues. We can say that these multifaceted perspectives show that the students are highly sensitive to global issues and have a solution-oriented perspective on these issues. It can be said that the participants' answers are similar to the results of the Global Issues Survey conducted by Villars

Institute [81] with more than 150 young people. In addition, the results of the Global Shapers Annual Survey [82] contain similar findings. It can be said that students made similar statements about global issues in the research of Akhan, Demirezen, and Çiçek [83]. In addition, it was seen that participants explained global issues in a similar way in the study of [84].

In this study, it can be seen that students are aware of sustainability and have a wide perspective from individual steps to global policies. It can be said that the participants attach importance to daily practices such as efficient use of resources, recycling, waste management and sustainable consumption habits and explain sustainability mostly in terms of environmental and social factors. In a similar way in the literature, Behm's [85] study includes similar findings. In the study of Owens, and Legere [86], students explained sustainability through more concrete and action-based practices. In addition, it can be said that in the study of Demirci and Teksöz [87], students' answers indirectly mentioned the economic and social aspects of sustainability in the environmental dimension.

When the actions taken by the students in the study group in their daily lives for a sustainable world are examined, it is seen that they carry out actions such as waste management and recycling, and the protection of resources. It can be said that the students in the study group are aware of their individual responsibilities and tend to produce solutions for global issues. Similarly, we can say that the preschool teachers in the study of [88] also focused on similar practices in their sustainability practices. Piscitelli and D'Uggento [89] examined the sustainable behaviours of young people through a survey conducted with high school students in their study and revealed that the participants had similar practices in recycling and the protection of resources. Similarly, Salonen, Siirilä and Valtonen [90] revealed that the participants had actions in the direction of recycling, waste management, and the protection of resources.

The fact that the international university students in the study group took into consideration both positive aspects such as ethical practices, cooperation and awareness and difficulties such as resource inequality and economic problems when evaluating the relationship between global citizenship and sustainability shows that they have a multifaceted perspective. In his study with university students, Bulut [64] revealed that individuals with high levels of global citizenship also have high levels of sustainable development.

When the decisions and actions they would make/take if they were UN secretaries are examined in the research, it can be said that the students can effectively take responsibility in areas such as climate action, education, social equality and innovative solutions. Their ability to address environmental and social dimensions in a balanced way reveals that they have a comprehensive global perspective. The findings show that the students have developed a solution-oriented approach to global problems and that this approach will shape their future understanding of global citizenship. It is possible to say that this approach of the students are compatible with the suggestions presented in response to today's global challenges in the document titled "Our Common Agenda" prepared by the United Nations Secretary-General [91] and the action plan prepared for the common well-being of humanity.

When the qualitative findings of the study are evaluated in general, it can be said that international university students can explain the concept of global citizenship and develop a global citizenship understanding, the majority of the participants see themselves as global citizens, they can explain the knowledge, skills and values that a global citizen should have, they have a broader perspective on global problems, they are sensitive to problems on both an individual and global scale, they have a collective responsibility for solving environmental and social problems, they have a global citizenship awareness and put this awareness into practice in their daily lives, they can comprehensively explain the impact of global citizenship on sustainability and they have a strong sustainability awareness and global citizenship

understanding. It is possible to state that these findings indicate that the participants who develop sensitivity to environmental and social problems approach global problems with a solution-oriented perspective and that this understanding will shape their future global citizenship potential.

Conclusions

- Interpretations are occasionally speculative and not fully supported by the data.
- Need to acknowledge study limitations (e.g., sample size, potential biases).

When the quantitative and qualitative findi[illegible]possible to say that international students have g[illegible]hile the quantitative findings define this awareness at a moderate level, the qualitative findings show that this awareness is higher and that students can evaluate the concept in multiple ways. In addition, both quantitative and qualitative findings show that students develop sensitivity to environmental and social problems. While the effect of social media on this awareness is emphasized in the quantitative findings, the qualitative findings associate this sensitivity with individual skills such as empathy, communication and ethical sensitivity. While the quantitative findings indicate that participation in global activities and membership in global organizations increase global citizenship tendencies, the qualitative findings point to the importance of solidarity and cooperation in this process. Both quantitative and qualitative findings agree that the active participation of individuals increases awareness.

When all the findings of the research are evaluated in general, it is revealed that global citizenship awareness is shaped in line with the geographical, cultural and social contexts in which individuals live, but that a universal understanding of global responsibility is increasingly gaining importance. Global citizenship goes beyond being a mere theoretical concept and enables individuals, especially students, to take a more active role in social and environmental issues.

As a result, global citizenship offers an understanding that allows individuals to approach the problems they face locally and globally in a more conscious and sensitive way.

Furthermore, as Torres [92] stated, sustainable development is the twin brother of global citizenship education. If we live in the century of sustainability and want to achieve the seventeen global goals, we must achieve the goals of sustainability and global citizenship together.

According to the Global Peace Index, there was a 0.56% decrease in peace levels worldwide in 2024 [93]. The theme of the 2024 G20 Brazilian Presidency, "Building a just world and a sustainable planet", is almost a response to the decreasing peace levels. The dissemination of the concept of global citizenship will contribute to global cooperation in building a sustainable future and to individuals having a global citizenship awareness and building a more peaceful world. In this context, we can say that global citizenship is an understanding that all individuals should take as a guide on the way to building a more just and sustainable world.

Limitations and Recommendations

The most obvious limitation of this study is that the participants in the qualitative interviews could not be students from different geographical regions. It would have been more explanatory for the research results to reveal that global citizenship tendencies differ according to regions such as Europe, Asia, and Sub-Saharan Africa through qualitative interviews. In addition, although the quantitative findings in the study revealed that age and social media are determinants of global citizenship tendencies and that gender has a minimal effect, these data could not be confirmed in the qualitative interviews. Because only students who could spare time for the interviews were included. This made it difficult to look at the variables in question in the qualitative interviews. In addition, while the qualitative data revealed that students experienced conflicts between global and national identities, this important information could not be examined in depth. In addition, there were no international students from America, Australia, or developed countries in Asia in the study group. This situation was due to the fact

that there were few students from these regions in the universities where the study was conducted and that they did not accept the offer of employment.

In line with the results of the research, it can be suggested that for future research, first of all, interviews should be conducted with students from different geographical regions mentioned in the limitations, these findings should be examined by conducting interviews in order to compare the findings regarding the variables revealed in the quantitative findings, the conflicts between the global identity and national identity of the students revealed in the qualitative findings should be examined in depth, and the results should be expanded for discussion by reaching out to students from different regions that could not be reached.

According to the research results, international university students have a moderate level of global citizenship awareness, but qualitative findings show that this awareness is at a higher level and that students can evaluate the concept in a multifaceted way. This result shows that students have the potential to reach higher levels. For students to reveal this potential, it can be thought that factors such as lack of information, national identity and global identity conflict can further increase the level of global citizenship awareness and responsibility with educational interventions, and students can take on a more active role in global problems. With educational support and awareness programs, these students can be enabled to adopt their global citizenship identities more effectively and develop more concrete actions towards sustainability goals. In this direction, studies testing the educational intervention in question can be recommended. In addition, work environments can be created where education, intercultural interaction, effective use of digital tools such as social media and experiential learning opportunities are created to support international university students.

International university students have a unique opportunity to develop intercultural interaction and global citizenship tendencies through their educational environments. Factors such as social media, global events and membership in global organizations are seen to

strengthen these tendencies. Providing more education, collaboration and technological support to increase students’ tendencies on the subject can help them put this awareness into action in daily life. Supporting this group with their young and dynamic structure to reduce inequalities and raise awareness to a global level is critical to creating a more conscious global society in the long term.

The results of this research reveal the necessity of strategic interventions to increase students' global citizenship awareness in education policies and global cooperation processes. Research to be conducted in this direction will contribute to the perception of global citizenship for a sustainable world.

Author Contributions

A.U., research administration, supervision, validation; O.A., conceptualization, data curation, methodology, software, writing—original draft, writing—review and editing; S.T., investigation, resources, writing—original draft, writing—review and editing; A.Ö., methodology, validation, writing—review and editing. All authors have read and agreed to the published version of the manuscript.

Funding

This research received no external funding.

Data Availability Statement

The original contributions presented in the study are included in the article; further inquiries can be directed to the corresponding author.

Conflicts of Interest

The author declares that the research was conducted in the absence of any commercial or financial relationship that could be construed as a potential conflict of interest.

References

1. Çelik MY. Boyutları ve farklı algılarıyla küreselleşme. Dumlupınar Üniversitesi Sosyal Bilimler Dergisi. 2012;32(2):57–74.

2. Barrow CW, Keck M. Globalization theory and state theory: The false antinomy. Studies in Political Economy. 2017;98(2):177–96.

3. Giddens A. Elimizden kaçıp giden dünya: Küreselleşme hayatımızı nasıl yeniden şekillendiriyor? 2000.

4. Robertson R. Küreselleşme toplum kuramı ve küresel kültür. Yolsal ÜH, Çev), editors. Ankara: Bilim ve Sanat Yayınları; 1999.

5. Akhan NE, Kaymak B. Sosyal bilgiler öğretmenlerinin küreselleşme, küresel eğitim ve küresel vatandaş kavramlarına yönelik görüşleri. Milli Eğitim Dergisi. 2021;50(229):129–55.

6. Volle A. globalization. Encyclopedia Britannica; 2024. Available from: https://www.britannica.com/money/globalization

7. Rizvi F. Globalization and the neoliberal imaginary of educational reform [Internet]. Paris: UNESCO; 2017. (Education Research and Foresight Series). Available from: https://en.unesco.org/node/262287

8. Leduc R. Global Citizenship Instruction Through Active Participation: What Is Being Learned About Global Citizenship? The Educational Forum [Internet]. 2013 Oct [cited 2024 Nov 1];77(4):394–406. Available from: http://www.tandfonline.com/doi/abs/10.1080/00131725.2013.822038

9. UNESCO. Global citizenship education: Topics and learning objectives [Internet]. 2015. Available from: https://unesdoc.unesco.org/.

10. UNICEF. Global citizenship: A high school educator's guide. 2013.

11. Oxfam Development Education Programme. Education for global citizenship: A guide for schools. Oxfam GB; 2006.

12. Sassen S. Towards post-national and denationalized citizenship. In: Isin EF, Turner BS, editors. Handbook of citizenship education. Sage; 2002. p. 277–91.

13. Castles S, Davidson A. Citizenship and migration: Globalization and the politics of belonging. Routledge; 2000.

14. UNESCO. What you need to know about global citizenship education [Internet]. 2024 [cited 2024 Nov 1]. Available from: https://www.unesco.org/en/global-citizenship-peace-education/need-know

15. Chakraborty I, Maity P. COVID-19 outbreak: Migration, effects on society, global environment and prevention. Science of the total environment [Internet]. 2020 [cited 2024 Nov 1];728:138882. Available from: https://www.sciencedirect.com/science/article/pii/S0048969720323998

16. Whitworth J. COVID-19: a fast evolving pandemic [Internet]. Vol. 114, Transactions of the Royal Society of Tropical Medicine and Hygiene. Oxford University Press; 2020 [cited 2024 Nov 1]. p. 241–8. Available from: https://academic.oup.com/trstmh/advance-article-pdf/doi/10.1093/trstmh/traa025/33012905/traa025.pdf

17. Kešeljević A, Spruk R. Estimating the effects of Syrian civil war. Empir Econ [Internet]. 2024 Feb [cited 2024 Nov 1];66(2):671–703. Available from: https://link.springer.com/10.1007/s00181-023-02470-2

18. Bennett AC, Knauer J, Bennett LT, Haverd V, Arndt SK. Variable influence of photosynthetic thermal acclimation on future carbon uptake in Australian wooded ecosystems under climate change. Global Change Biology [Internet]. 2024 Jan [cited 2024 Nov 1];30(1):e17021. Available from: https://onlinelibrary.wiley.com/doi/10.1111/gcb.17021

19. Burton C, Lampe S, Kelley DI, Thiery W, Hantson S, Christidis N, et al. Global burned area increasingly explained by climate change. Nature Climate Change [Internet]. 2024;1–7. Available from: https://www.nature.com/articles/s41558-024-02140-w

20. Gajendiran K, Kandasamy S, Narayanan M. Influences of wildfire on the forest ecosystem and climate change: A comprehensive study. Environmental Research [Internet]. 2024 [cited 2024 Nov 1];240:117537. Available from: https://www.sciencedirect.com/science/article/pii/S0013935123023411

21. Hanna E, Topál D, Box JE, Buzzard S, Christie FD, Hvidberg C, et al. Short-and long-term variability of the Antarctic and Greenland ice sheets. Nature Reviews Earth & Environment [Internet]. 2024 [cited 2024 Nov 1];5(3):193–210. Available from: https://www.nature.com/articles/s43017-023-00509-7

22. Dobson A. Citizenship and the environment. Oxford University Press; 2003.

23. Thiele LP. Sustainability. John Wiley & Sons; 2024. 152 p.

24. United Nations. Transforming our world: the 2030 Agenda for Sustainable Development | Department of Economic and Social Affairs [Internet]. 2015 [cited 2024 Nov 1]. Available from: https://sdgs.un.org/2030agenda

25. Feil AA, Schreiber D. Sustainability and sustainable development: Unraveling overlays and scope of their meanings. Cadernos EBAPE BR. 2017;14(3):667–81.

26. Sağdıç A. A closer look into Turkish elementary teachers regarding education for sustainable development. [Ankara]: Middle East Technical University, Institute of Social Sciences; 2013.

27. Akcay K, Altinay F, Altınay Z, Daglı G, Shadiev R, Altinay M, et al. Global citizenship for the students of higher education in the realization of sustainable development goals. Sustainability. 2024;16(1604).

28. United Nations. Annual report 2020: A year in review. UNDP [Internet]. 2020. Available from: https://annualreport.undp.org/2020/assets/UNDP-Annual-Report-2020-en.pdf

29. Radhakrishna RB, Ingram PD. Experiences of 4-H Japanese exchange program on participants: An evaluative study. Journal of Extension [Internet]. 2005;43(3). Available from: https://joe.org/joe/2005june/rb3.php

30. Cotton DRE, Morrison D, Magne P, Payne S, Heffernan T. Global citizenship and cross-cultural competency: Student and expert understandings of internationalization terminology. Journal of Studies in International Education. 2019;23(3):346–64.

31. Lockett L, Moore L, Wingenbach G. A global worldview among extension professionals: A case study of best practices for study abroad programs. Journal of Extension [Internet]. 2014;52(4). Available from: https://joe.org/joe/2014august/a3.php

32. Gibson KL, Rimmington GM, Landwehr-Brown M. Developing global awareness and responsible world citizenship with global learning. Roeper Review. 2008;30(1):11–23.

33. Bourn D. Global citizenship and youth participation. 2016 [cited 2024 Nov 5]; Available from: https://discovery.ucl.ac.uk/id/eprint/1475443/

34. Morse JM, Maddox LJ. Analytic integration in qualitatively driven (QUAL) mixed and multiple methods design. In: Flick U, editor. The Sage handbook of qualitative data analysis. Sage Publications; 2014. p. 524–39.

35. Creswell JW, Plano Clark VL, Gutmann ML, Hanson WE. Advanced mixed methods research designs. In: Tashakkori A, Teddlie C, editors. Handbook of mixed methods in social and behavioral research. Sage Publications; 2003. p. 209–40.

36. Creswell JW. Research design: Qualitative, quantitative, and mixed methods approach. 4th ed. Sage Publications; 2014.

37. Reysen S, Katzarska-Miller I. A model of global citizenship: Antecedents and outcomes. International Journal of Psychology. 2013;48(5):858–70.

38. The jamovi project [Internet]. 2023. Available from: https://www.jamovi.org.

39. George D, Mallery P. Descriptive statistics. In: IBM SPSS Statistics 26 Step by Step: A Simple Guide and Reference. 16th ed. Routledge; 2020. p. 112–20.

40. Tabachnick BG, Fidell LS. Using multivariate statistics. 6th ed. Pearson; 2012.

41. Kerby DS. The Simple Difference Formula: An Approach to Teaching Nonparametric Correlation. Comprehensive Psychology [Internet]. 2014 Jan [cited 2024 Jul 15];3:11.IT.3.1. Available from: http://cop.sagepub.com/lookup/doi/10.2466/11.IT.3.1

42. Lacobucci D, Popovich DL, Moon S, Román S. How to calculate, use, and report variance explained effect size indices and not die trying. Journal of Consumer Psychology. 2023;33(1):45–61.

43. Woods M, Paulus T, Atkins DP, Macklin R. Advancing qualitative research using qualitative data analysis software (QDAS): Reviewing potential versus practice in published studies using ATLAS.ti and NVivo, 1994–2013. Social Science Computer Review. 2016;34(5):597–617.

44. Engin G, Sarsar F. Investigation of primary school teacher candidates' global citizenship levels. Journal of Human Sciences. 2015;12(1):150–61.

45. Kayışoğlu NB. Investigation of global citizenship levels of pre-service physical education teachers. Educational Research and Reviews. 2016;11:299–306.

46. Karatekin K, Taban MH. Global citizenship levels of Polish university students and Turkish Erasmus students in Poland. Acta Didactica Napocensia. 2018;11(1):41–59.

47. Özdemir Özden D, Karadağ Y. Sosyal bilgiler öğretmen adaylarının küresel vatandaşlık düzeylerinin incelenmesi. International Journal of Field Education. 2021;7(2):12–35.

48. Gerodimos R, Balbin C, Chan C, Freundt-Thurne U, Gutiérrez Atala FJ, Nyaole-Kowuor R, et al. Global citizenship in comparative perspective: Youth perceptions of global rights, responsibilities, and efficacy across five continents. Observatorio (OBS) Journal. 2022;

49. Alsawi AAH. Global citizenship skills among Qatar University students. Humanities and Social Sciences Communications. 2023;10(698).

50. Biay KKL, Tenorio AC. The relationship of cultural intelligence and global citizenship levels of preservice teachers in the Philippines. International Journal of Contemporary Educational Research. 2024;11(3):387–406.

51. Liu CL, Chung ML, Hui BPH, Li HC. The effect of social capital on outcomes of global citizenship among Taiwanese young adults: The mediating role of political self-efficacy. Sage Open. 2023;13(1).

52. Szelenyi K, Rhoads RA. Citizenship in a global context: The perspectives of international graduate students in the United States. Comparative Education Review. 2007;51(1):25–47.

53. Der-Karabetian A, Cao Y, Alfaro M. Sustainable behavior, perceived globalization impact, world-mindedness, identity, and perceived risk in college samples from the United States, China, and Taiwan. Ecopsychology. 2014;6:218–33.

54. Katzarska-Miller I, Reysen S, Kamble SV, Vithoji N. Cross-national differences in global citizenship: Comparison of Bulgaria, India, and the United States. Journal of Globalization Studies. 2012;3(2):166–83.

55. Cleofas JV, Labayo CC. Youth netizens as global citizens: Digital citizenship and global competence among undergraduate students. Frontiers in Communication. 2024;9, Article 1398001.

56. Ekici Ö. Impact of social media on global citizenship according to social studies teachers. International Journal of Modern Education Studies. 2023;7(2):457–74.

57. Ben Ltaifa M, Derbali AMS. The importance of social networks in enhancing the dimensions of citizenship among students in Saudi Arabia. Education Research International. 2022;2990659.

58. Lee RB, Baring R, Maria MS, Reysen S. Attitude towards technology, social media usage, and grade-point average as predictors of global citizenship identification in Filipino university students. International Journal of Psychology. 2017;52(3):213–9.

59. Çakmak Z, Bulut B, Taşkiran C. A study on the relationship between global citizenship perceptions and internet use of social studies pre-service teachers. Adıyaman University Journal of Educational Sciences. 2015;5(1):99–112.

60. Maguth BM, Yamaguchi M. The use of social networks in the social studies for global citizenship education: Reflecting on the March 11, 2011 disaster in Japan. The Georgia Social Studies Journal. 2013;3(2):80–93.

61. Kaya MM. The relationships between global citizenship, multicultural personality, and critical thinking. Educational Policy Analysis and Strategic Research. 2021;16(4):274–92.

62. Celume MP, Maoulida H. Developing 21st century competencies among youth through an online learning program: BE a global citizen. Education Sciences. 2022;12(3):148.

63. Chui WH, Leung EWY. Youth in a global world: Attitudes towards globalization and global citizenship among university students in Hong Kong. Asia Pacific Journal of Education. 2014;34(1):107–24.

64. Bulut B. Correlation between global citizenship and sustainable development awareness levels of pre-service teachers. International Online Journal of Educational Sciences. 2019;11(3):279–93.

65. Sherman PD. The cultivation and emergence of global citizenship identity. Citizenship Teaching & Learning. 2019;14(1):7–25.

66. Smith WC, Fraser P, Chykina V, Ikoma S, Levitan J, Liu J, et al. Global citizenship and the importance of education in a globally integrated world. Globalisation, Societies and Education. 2017;15(5):648–65.

67. Reysen S, Hackett J. Activism as a pathway to global citizenship. The Social Science Journal. 2017;54(2):132–8.

68. Troll T. Another world is happening: Towards a great transition through a global citizens movement (Master’s dissertation. Faculty of Culture and Pedagogy, Institute of Education, University of London; 2014.

69. Albanesi C, Guarino A, Zani B, Cicognani E, Tzankova I. Civic engagement in a changing world: Does it contribute to the development of global citizenship? TPM-Testing, Psychometrics. Methodology in Applied Psychology. 2019;26(4):495–511.

70. Aydin H, Andrews K. U.S. and Mexican college students' perceptions of global citizenship education: A comparative case study. Globalisation, Societies and Education. 2024;

71. Aydin H, Ogurlu U, Andrews K, Masalimaova A, Dorozhkin E, Malygin A. High school students' perceptions of global citizenship in central public high schools: Implications for teacher educators. Review of Research and Social Intervention. 2019;65(1):187–205.

72. Altıkulaç A, Yontar A. Nationalism, patriotism, and global citizenship: A comparison between social studies teacher candidates in the US and Turkey. International Journal of Education and Literacy Studies. 2019;7(4):115–23.

73. Kuleta-Hulboj M. The global citizen as an agent of change: Ideals of the global citizen in the narratives of Polish NGO employees. Journal for Critical Education Policy Studies. 2016;14(3):220–50.

74. Massey K. Global citizenship education in a secondary geography course: The students' perspectives. Review of International Geographical Education Online. 2014;4(2):80–101.

75. Inglehart R, Haerpfer C, Moreno A, Welzel C, Kizilova K, Diez-Medrano J, et al. World values survey: Round six – country-pooled datafile version [Internet]. 2014. Available from: www.worldvaluessurvey.org/WVSDocumentationWV5.jsp

76. Baker W, Fang F. So maybe I'm a global citizen': developing intercultural citizenship in English medium education. Language, Culture and Curriculum. 2021;34(1):1-17,.

77. Altıkulaç A. Patriotism and global citizenship as values: A research on social studies teacher candidates. Journal of Education and Practice. 2016;7(36):26–33.

78. Günel E, Pehlivan A. Pre-service social studies teachers' perception of global citizenship. Journal of Education and Future. 2016;10:51–69.

79. Oxfam. Education for global citizenship: A guide for schools. Oxfam Development Education; 2015.

80. Hunter WD. Knowledge, skills, attitudes, and experiences necessary to become globally competent (Doctoral dissertation. Pennsylvania: Lehigh University; 2004.

81. Institute TV. Global Issues Survey 2023 [Internet]. Villars Institute; 2023. Available from: https://villarsinstitute.org/posts/global-issues-survey-2023

82. Forum WE. Global Shapers Survey 2017 [Internet]. Amnesty International; 2017. Available from: https://www.es.amnesty.org/fileadmin/noticias/ShapersSurvey2017_Full_Report_24Aug__002__01.pdf

83. Akhan NE, Çiçek S, Kocaağa G. Critical and creative perspectives of gifted students on global problems: Global climate change. Thinking Skills and Creativity. 2022;46, Article 101015.

84. García-González E, Jiménez-Fontana R, Azcárate Goded P. Approaches to teaching and learning for sustainability: Characterizing students' perceptions. Journal of Cleaner Production. 2020;274:122928.

85. Behm CL. University of Illinois; 2011.

86. Owens KA, Legere S. What do we say when we talk about sustainability? Analyzing faculty, staff, and student definitions of sustainability at one American university. International Journal of Sustainability in Higher Education. 2015;16(3):367–84.

87. Demirci S, Teksöz G. Self-efficacy beliefs on integrating sustainability into profession and daily life: In the words of university students. International Electronic Journal of Environmental Education. 2017;7(2):116–33.

88. Tarı A, Mercan Uzun E. Okul öncesi öğretmenlerinin çevresel sürdürülebilirlik hakkındaki görüşlerinin ve uygulamalarının incelenmesi. Milli Eğitim Dergisi. 2023;52(240):3139–60.

89. Piscitelli A, D'Uggento AM. Do young people really engage in sustainable behaviors in their lifestyles? Social Indicators Research. 2022;163(3):1467–85.

90. Salonen AO, Siirilä J, Valtonen M. Sustainable living in Finland: Combating climate change in everyday life. Sustainability. 2018;10(1):104.

91. United Nations. Our common agenda: Report of the Secretary-General [Internet]. 2021. Available from: https://www.un.org

92. Torres CA. Global citizenship education and sustainability as real utopias. In: Bourn D, Tarozzi M, editors. Pedagogy of hope for global social justice: Sustainable futures for people and the planet. Bloomsbury Academic; 2024. p. 13–29.

93. Economics I, Peace. Global Peace Index 2024: Measuring peace in a complex world [Internet]. Institute for Economics & Peace; 2024. Available from: http://visionofhumanity.org/resources.
