# Supplementary material for: Determining the global citizenship tendencies of international university students for a sustainable world
Source: PLoS One. 2025 Oct 31;20(10):e0335817. doi: 10.1371/journal.pone.0335817 (PMC12578196; doi:10.1371/journal.pone.0335817)
Supplement: Attachment — Submitted filename: PONE-Uzlifatul-reviewer.pdf [file pone.0335817.s004.pdf]

The followings are some comments of the manuscript entitled "Determining the Global Citizenship Tendencies of International University Students for a Sustainable World" . The comments cover the strengths, suggestions, and the overall evaluation.

Title. The title is acceptable. It definitely reflects the core focus of the research: global citizenship tendencies of international students and sustainability. It is recommended to consider shortening it slightly for conciseness, e.g., *"Global Citizenship Tendencies of International Students for a Sustainable World"*, though the current version is still acceptable.

Abstract. The abstract has summarized the mixed-methods approach and key findings well, indicating a moderate level of global citizenship awareness among students. However, It is better to add specific numerical results or statistical findings for quantitative clarity. Moreover, Briefly state the implications of the findings to give a more complete overview.

Introduction. The introduction part provides a compelling rationale by connecting global challenges with the need for global citizenship. The link between education, intercultural understanding, and sustainability is well articulated. However, it is better to strengthen the justification for the focus on Turkey and why international students there are especially relevant. Cite more recent empirical studies related to global citizenship trends.

Literature Review. The literature review covers major conceptual models and prior empirical findings regarding global citizenship, sustainability, and student identity. It is effectively frames global citizenship within the SDGs (especially Goals 4 and 16).

Method. The Mixed-methods design is appropriate and well justified. Use of validated tools (e.g., Global Citizenship Scale) and MAXQDA for qualitative data strengthens reliability. Details on normality testing and appropriate nonparametric analysis methods are included. However, it is suggested to provide more details could be provided on the sampling procedure and demographics as well as justify why certain statistical tests were chosen over others beyond referencing skewness/kurtosis.

Findings. The quantitative results show significant relationships between global citizenship and variables such as age, global event participation, and association membership. The qualitative results add richness by capturing students' nuanced perspectives, such as hypothetical actions as UN Secretary General. It is suggested to integrate findings from both methods more cohesively throughout the results, not just in the discussion and highlight specific themes from qualitative responses using tables or diagrams.

Discussion. In discussion, strong interpretation of how quantitative and qualitative findings complement each other. It emphasizes educational and policy implications, e.g., curricular integration and social media's role in shaping global citizenship. It is recommended to while

contextualization is good, more critical reflection on contradictory findings (e.g., gender effects) would enhance depth. Further, it is better to discuss the potential for causality cautiously since this is largely a correlational study.

Conclusion. It clearly restates key findings and advocates for strategic educational interventions. This suggests future directions, such as educational programs to enhance awareness and global responsibility. .

Suggestions. The suggestions could be more concise and acknowledge limitations more directly in this section instead of relying solely on the separate limitations section.

Limitations show its transparent about regional sample limitations and lack of participant diversity from areas like the Americas or East Asia. However, it is suggested to discuss how these limitations may have influenced the results or generalizability more directly.

References The reference list is extensive and includes both foundational and recent studies relevant to global citizenship, sustainability, and education. Hopefully, it is recommended to ensure uniform formatting and inclusion of DOIs where possible. Also, a few references could be updated or replaced with more recent ones from 2022–2024.

Overall Evaluation

Strengths of the manuscript are It is a well-structured, methodologically sound, and timely study. In addition, it addresses a critical global issue using robust analytical tools and diverse data sources.

Recommendations for Improvement: The manuscript should be enhanced with integration of mixed methods, more concise and critically reflective discussion and conclusion. Also broaden sample diversity in future research and acknowledge regional contextual influences more thoroughly.

Reviewer Comments Overall Evaluation

This manuscript titled *"Determining the Global Citizenship Tendencies of International University Students for a Sustainable World"* presents a timely and relevant study investigating global citizenship awareness among international students in Turkey using a robust mixed-methods approach. The study is well-aligned with global education and sustainability agendas, particularly the SDGs.

The Strengths of the manuscript are explained as follows: The study uses a **convergent parallel mixed-methods design** effectively, combining a validated Global Citizenship Scale and rich qualitative interviews. The **sample size (N=634)** is adequate and demographically diverse

within the context of Turkey. The **quantitative analyses** are appropriately selected, with careful attention to normality assumptions and non-parametric tests. The **qualitative findings** offer nuanced insights into student perceptions, identities, and values. The manuscript makes **practical recommendations** for integrating global citizenship education in higher education policy and curriculum.

Major Comments

1. **Integration of Mixed Methods**: The manuscript would benefit from more clearly integrated discussion of how qualitative and quantitative findings complement or contrast with each other throughout the discussion section.
2. **Generalizability**: The authors acknowledge that participants are all from universities in Turkey, but further discussion on how cultural or institutional contexts may limit generalizability would strengthen the conclusion.
3. **Critical Synthesis of Literature**: The literature review is comprehensive but could be improved with more critical engagement and synthesis rather than descriptive listing.
4. **Clarity in Results Presentation**: The quantitative results section is dense. Including summary tables or visualizations to highlight significant findings by key variables (e.g., gender, age, participation in global events) would improve readability.

Minor Comments

- The **abstract** should briefly include key quantitative results (e.g., mean scores or significant differences).
- Some **references** could be updated with more recent sources from the last two years.
- Ensure **uniform formatting** of references and correct minor grammatical issues.

Recommendation : Minor Revision — The manuscript presents high-quality research with important implications for global education and sustainability. With some revisions to improve clarity, integration of findings, and critical framing, it would make a valuable contribution to the literature.
