# Supplementary material for: Determining the global citizenship tendencies of international university students for a sustainable world
Source: PLoS One. 2025 Oct 31;20(10):e0335817. doi: 10.1371/journal.pone.0335817 (PMC12578196; doi:10.1371/journal.pone.0335817)
Supplement: Attachment — Submitted filename: Revision Text.docx [file pone.0335817.s002.docx]

**ABSTRACT**

1. The opening sentence is too general, Sentence: "This study examines the global citizenship tendencies of international university students and the direction of these tendencies towards a sustainable world."
2. Problem: Too broad and does not directly state the specific purpose of the study.
3. Suggestion: Specify that the focus is on the relationship between students' global awareness and enrollment practices.
4. The term "international university" is ambiguous, This term can be interpreted in various ways: is it a university with an international curriculum? Or international students at any university?

-Suggestion: Use the term "international students" (international students) consistently, and define them as studying at universities in Turkey.

5. Redundancy and imprecision of sentences, Sentence: "When the results of the study were evaluated as a whole, the quantitative findings showed that global awareness was at a moderate level, while the qualitative findings showed that this awareness was higher..."

- Problem: Too long, not sharp enough, and seems repetitive.

- Suggestion: Split into two sentences and summarize the language to make it more academic.

6. Inconsistent and overly narrative language, Example: "Both quantitative and qualitative findings coincide..."

- In academic writing, it is better to use terms such as “converged” or “revealed same pattern.

7. Conclusion needs to be strengthened. The last sentence can clarify the research contribution and intended policy recommendations.

**INTRODUCTION**

General Notes Required Revisions:

1. Too Long and Not Clearly Structured, This introduction contains too much information at once without clear paragraph divisions or subtopics. This makes it difficult for readers to grasp the logical flow of the research problem.

- Suggestion: Divide the introduction into 4–6 main paragraphs, each with the following focus:

a. The context of globalization and the challenges of today's world

b. The role of global citizenship in responding to these challenges

c. The relationship between global citizenship and sustainability

d. The role of international students in this context

e. The purpose and contribution of the study

2. Too Narrative and Not Academic Style, Some parts sound like essays or popular articles, not scientific writing.
   a. Example sentence: "What does it mean to be a global citizen? Is it just about knowing the capital city, finding famous places..."

- Suggestion: Eliminate the question-and-answer style and use descriptive-analytical language. For example: Being a global citizen is not only about understanding geography and culture, but also about actively participating in cross-border issues.

3. Several Paragraphs Deviate from the Research Focus, The explanation of the definition of globalization and its history is too long, even though not all of it is directly relevant to the research focus.

- Suggestion: Summarize the theory of globalization section, just 2-3 main definitions that are directly relevant to global citizenship.

4. The Use of Lists or Images (such as Figure 1) is Ineffective, Figure 1 which explains the components of global citizenship is not displayed and is not well integrated into the narrative.

-Suggestion: If you include visuals, make sure they are integrated narratively and relevantly. If not crucial, it should be removed from the introduction.

5. The Research Objective Paragraph is Unclear and Hidden, The study objectives only appear at the very end and are not directly stated explicitly.

- Suggestion: Add an objective paragraph at the end of the introduction that directly explains:

a. What is the purpose of this study

b. Why is it important

c. What gaps do you want to fill

-Example of Recommended Introduction Structure:

1. Paragraph 1: Global Context

a. Global change after the 20th century

b. Global challenges: inequality, climate crisis, social conflicts

2. Paragraph 2: The Role of Global Citizenship

a. Definitions and importance of the concept from various organizations

b. Relation to diversity, solidarity, and global justice

3. Paragraph 3: Global Citizenship and Sustainability

a. Direct relationship to the Sustainable Development Goals (SDGs)

b. Goals 4 and 16 as key examples

4. Paragraph 4: International Students as Agents of Change

a. International mobility → intercultural interaction → global awareness

b. Students as future leaders

5. Paragraph 5: Research Gap

a. Lack of research on how international students internalize global citizenship

6. Paragraph 6: Research Objectives

a. “This study aims to explore the understanding and application of global citizenship among international students in Turkey and their contributions to sustainable practices.

**METHOD**

1. Structure and Naming of Subsections

Problem: Not all methods sections are structured with consistent subheadings. Some sections are quite long and dense.

Revision: Separate the methods into consistent and structured subheadings, for example:

• Research Design

• Study Participants

• Data Collection Procedures

• Ethical Considerations

• Instruments

• Quantitative Data Analysis

• Qualitative Data Analysis

• Trustworthiness (for validity and reliability of qualitative data)

2. Explanation of Mixed Models is Not Clear

Problem: Explanation of the "convergent parallel mixed design" model is still repetitive and not efficient.

3. Description of Participants Needs to be Refined

Problem: Description of country of origin is too detailed without further meaning, and could be condensed.

4. Redundancy in Data Collection

Problem: Explanation of data collection via email, WhatsApp, Google Form, etc., is too long. Summarize concisely and formally

5. Instrument Description Too Detailed

Problem: Explaining Scale items one by one in an unconventional method; this is better suited to an appendix. Simplify

6. Statistical Analysis Explanation Needs Focus

Problem: Too much technical detail (such as p-values for each normality test) is not all necessary in the method.

7. Validity and Reliability of Qualitative Data

Problem: Explanation is too narrative and lacks reference to theory or references. Add references and make it systematic.

**CONCLUSIONS**

1. Clarification and Sharpening of Synthesis of Findings

Problem: Some sentences are still too general or narrative, without emphasizing the scientific contribution explicitly.

Solution: Use more concise and specific academic language.

Example: Quantitative findings indicate a moderate level of global citizenship awareness, while in-depth interviews reveal a more complex and reflective understanding of the concept, including aspects of empathy, solidarity, and cross-cultural values.

2. Avoid Redundant or Overly Long Sentences

Problem: There are several sentences that are long and contain more than one idea.

Solution: Break them into several sentences to make them clearer and more academic.

Example: Global citizenship is not just a normative concept, but an operational framework that shapes students' active role in social and environmental issues.

3. Focus on Implications and Scientific Contributions

Problem: There is no specific paragraph that highlights the practical and theoretical implications of the findings.

Solution: Add a final paragraph that summarizes the contributions to the literature, policy, or educational practice.

Example: This study contributes to the global citizenship literature by combining quantitative and qualitative approaches in the context of international students. The implications are important for higher education curriculum designers who want to instill global citizenship values more holistically.

4. Avoid Abrupt Topic Shifts

Problem: Suddenly entering the G20 and the Global Peace Index without a strong transition.

Solution: Connect the global data with the findings more systematically.

Example: The current global context, such as the decline in the world peace index by 0.56% in 2024, shows the urgency of integrating global citizenship in education. The theme of the G20 Brazil 2024 also emphasizes that global citizenship education is the foundation for social resilience and sustainable development.

5. End with a Strong and Reflective Sentence

Problem: The final sentence is too normative.

Solution: Close with a reflective sentence and based on the contribution of this study.

Example: Thus, the integration of global citizenship values in higher education plays a strategic role in creating a generation that is not only locally aware, but also globally responsible.
