# Supplementary material for: Determining the global citizenship tendencies of international university students for a sustainable world
Source: PLoS One. 2025 Oct 31;20(10):e0335817. doi: 10.1371/journal.pone.0335817 (PMC12578196; doi:10.1371/journal.pone.0335817)
Supplement: Attachment — Submitted filename: Response to feedback.docx [file pone.0335817.s005.docx]

**Determining The Global Citizenship Tendencies of International University Students for a Sustainable World**

**Referee Recommendations and Author Disclosures**

| **REFEREE 1** | **AUTHORS** |
| --- | --- |
| Overall, the manuscript is well-written and presents a clear and relevant discussion. The objectives are well addressed, and the arguments are supported with appropriate references. However, the Conclusion section could be improved to better reflect the key findings and their implications. At present, it reads more like a summary rather than offering a strong final reflection or critical insight. I recommend revising this section to emphasize the main contributions of the study, highlight its significance, and, if applicable, suggest directions for future research. | The conclusion section has been edited according to your suggestion. |
| **Note:** Thank you for your valuable comments. We tried to make all your suggestions, and we hope they meet your expectations. | |

| **REFEREE 2** | **AUTHORS** |
| --- | --- |
| **1) I found a typographical word in lines 180-181. It should be "By integrating quantitative and qualitative findings...".** | Edited according to your suggestion. |
| **2) What methods did the authors employ to guarantee that the selection criteria—completion of at least one semester of education in Turkey with international student status and English language proficiency—were uniformly implemented across the four universities? Additionally, what steps were taken to confirm the students' English language proficiency?** | Three of these four universities are in the same city. The authors/faculty members at these universities contacted the students via email with the assistance of the international relations office. Students were emailed in English and asked about their English proficiency. Students were also informed in the email that the interviews would be in English. Students who accepted all the terms and responded to the invitation email were accepted into the study group. Another university is the university in the city where the first author resides. The first author also contacted the students in the study group in the same way with the assistance of the international relations office at his university.  This information has been added to the appropriate paragraphs within the article. |
| **3) I would recommend the authors to use thematic analysis as their main approach because international students bring diverse cultural perspectives that might not be fully captured by predetermined coding schemes. Thematic analysis focuses on interpretation, seeking to uncover the deeper meaning and significance within the data, whereas content analysis typically emphasizes quantifying how often specific elements appear. Thematic analysis is suitable for analyzing individual perspectives as seen in lines 238-239 that the authors aim to reveal participant perceptions.** | It was organized as a thematic analysis. |
| Note: Thank you for your valuable comments. We tried to make all your suggestions, and we hope they meet your expectations. | |

| **REFEREE 3** | **AUTHORS** |
| --- | --- |
| ABSTRACT  1. The opening sentence is too general, Sentence: "This study examines the global citizenship tendencies of international university students and the direction of these tendencies towards a sustainable world."  2. Problem: Too broad and does not directly state the specific purpose of the study.  3. Suggestion: Specify that the focus is on the relationship between students' global awareness and enrollment practices.  4. The term "international university" is ambiguous, this term can be interpreted in various ways: is it a university with an international curriculum? Or international students at any university?  -Suggestion: Use the term "international students" (international students) consistently and define them as studying at universities in Turkey.  5. Redundancy and imprecision of sentences, Sentence: "When the results of the study were evaluated as a whole, the quantitative findings showed that global awareness was at a moderate level, while the qualitative findings showed that this awareness was higher..."  - Problem: Too long, not sharp enough, and seems repetitive.  - Suggestion: Split into two sentences and summarize the language to make it more academic.  6. Inconsistent and overly narrative language  Example: "Both quantitative and qualitative findings coincide..."  - In academic writing, it is better to use terms such as “converged” or “revealed same pattern.”  7. Conclusion needs to be strengthened. The last sentence can clarify the research contribution and intended policy recommendations. | The opening and closing sentences were revised. The purpose was stated more clearly.  The purpose was stated more clearly:  The phrase "enrollment practices" was replaced with a link to sustainability.  The phrase "international university" was replaced with "international students studying at universities in Turkey."  Long and repetitive sentences were broken up.  "converged" was used instead of "coincide."  Based on your suggestion, the following questions are presented more clearly:  What is the purpose of this study? Why is it important? What gap does it aim to fill? |
|  |  |
| INTRODUCTION  General Notes Required Revisions:  1. Too Long and Not Clearly Structured, this introduction contains too much information at once without clear paragraph divisions or subtopics. This makes it difficult for readers to grasp the logical flow of the research problem.  - Suggestion: Divide the introduction into 4–6 main paragraphs, each with the following focus:  a. The context of globalization and the challenges of today's world  b. The role of global citizenship in responding to these challenges  c. The relationship between global citizenship and sustainability  d. The role of international students in this context  e. The purpose and contribution of the study  2. Too Narrative and Not Academic Style, some parts sound like essays or popular articles, not scientific writing.  Example sentence: "What does it mean to be a global citizen? Is it just about knowing the capital city, finding famous places..."  - Suggestion: Eliminate the question-and-answer style and use descriptive-analytical language. For example: Being a global citizen is not only about understanding geography and culture, but also about actively participating in cross-border issues.  3. Several Paragraphs Deviate from the Research Focus, the explanation of the definition of globalization and its history is too long, even though not all of it is directly relevant to the research focus.  - Suggestion: Summarize the theory of globalization section, just 2-3 main definitions that are directly relevant to global citizenship.  4. The Use of Lists or Images (such as Figure 1) is Ineffective, Figure 1 which explains the components of global citizenship is not displayed and is not well integrated into the narrative.  Suggestion: If you include visuals, make sure they are integrated narratively and relevantly. If not crucial, it should be removed from the introduction.  5. The Research Objective Paragraph is Unclear and Hidden, the study objectives only appear at the very end and are not directly stated explicitly.  - Suggestion: Add an objective paragraph at the end of the introduction that directly explains:  a. What is the purpose of this study  b. Why is it important  c. What gaps do you want to fill  -Example of Recommended Introduction Structure:  1. Paragraph 1: Global Context  a. Global change after the 20th century  b. Global challenges: inequality, climate crisis, social conflicts  2. Paragraph 2: The Role of Global Citizenship  a. Definitions and importance of the concept from various organizations  b. Relation to diversity, solidarity, and global justice  3. Paragraph 3: Global Citizenship and Sustainability  a. Direct relationship to the Sustainable Development Goals (SDGs)  b. Goals 4 and 16 as key examples  4. Paragraph 4: International Students as Agents of Change  a. international mobility → intercultural interaction → global awareness  b. Students as future leaders  5. Paragraph 5: Research Gap  a. Lack of research on how international students internalize global citizenship  6. Paragraph 6: Research Objectives  a. “This study aims to explore the understanding and application of global citizenship among international students in Turkey and their contributions to sustainable practices. | We have completely restructured the introduction section in alignment with your recommended thematic organization. The revised introduction now consists of the following logically ordered paragraphs:  Context of globalization and global challenges  The role and importance of global citizenship  The relationship between global citizenship and sustainability  The unique role of international students in this framework  The Turkish context and international student demographics  Research aim, significance, and contribution to literature.  The informal question-and-answer style has been entirely removed from the revised text. In its place, we have adopted a formal, analytical, and descriptive academic tone throughout the introduction.  We significantly condensed the explanation of globalization. The revised section now includes only the most relevant and widely cited definitions from the literature to establish the conceptual link between globalization and global citizenship.  We have removed Figure 1 from the introduction section. Instead, relevant conceptual components of global citizenship are now integrated directly into the text.  We reviewed the introduction, and it states the objective of the study, explains why it matters, clarifies the research gap |
|  |  |
| METHOD  1. Structure and Naming of Subsections  Problem: Not all methods sections are structured with consistent subheadings. Some sections are quite long and dense.  Revision:  Separate the methods into consistent and structured subheadings, for example:  • Research Design  • Study Participants  • Data Collection Procedures  • Ethical Considerations  • Instruments  • Quantitative Data Analysis  • Qualitative Data Analysis  • Trustworthiness (for validity and reliability of qualitative data)  2. Explanation of Mixed Models is Not Clear  Problem: Explanation of the "convergent parallel mixed design" model is still repetitive and not efficient.  3. Description of Participants Needs to be Refined  Problem: Description of country of origin is too detailed without further meaning and could be condensed.  4. Redundancy in Data Collection  Problem: Explanation of data collection via email, WhatsApp, Google Form, etc., is too long. Summarize concisely and formally  5. Instrument Description Too Detailed  Problem: Explaining Scale items one by one in an unconventional method; this is better suited to an appendix. Simplify  6. Statistical Analysis Explanation Needs Focus  Problem: Too many technical details (such as p-values for each normality test) are not all necessary in the method.  7. Validity and Reliability of Qualitative Data  Problem: Explanation is too narrative and lacks reference to theory or references. Add references and make it systematic. | We have reorganized the entire Methods section into the following subheadings, in accordance with your suggestions:  Research Design  Participants  Data Collection Procedures  Instruments  Ethical Considerations  Quantitative Data Analysis  Qualitative Data Analysis  Trustworthiness of Qualitative Data  We state that both data types were collected simultaneously, analyzed separately, and integrated during interpretation. Citations supporting the convergent parallel model were retained but redundant phrasing was removed for efficiency.  The regional grouping remains (Asia, Europe, etc.) because it is used in inferential analysis, but excessive enumeration was removed. The rationale for choosing students with English proficiency has also been summarized more concisely.  The data collection procedure has been revised and shortened.  **Explanations regarding the levels of individuals based on their scale scores are provided in the results and discussion section. Since informing the reader about these levels is considered important for the reading process of the article, this information has been included in the findings section.**  **In the study examining the normality of the data, decisions on the analyses were made based on the results of the Shapiro-Wilk normality test. In this context, it is considered more appropriate to present the results of the normality test. If the p-values are omitted, the reader may assume that normality was assessed based solely on skewness and kurtosis values.**  We have revised the trustworthiness procedures to align with established frameworks |
|  |  |
| CONCLUSIONS  1. Clarification and Sharpening of Synthesis of Findings  Problem: Some sentences are still too general or narrative, without emphasizing the scientific contribution explicitly.  Solution: Use more concise and specific academic language.  Example: Quantitative findings indicate a moderate level of global citizenship awareness, while in-depth interviews reveal a more complex and reflective understanding of the concept, including aspects of empathy, solidarity, and cross-cultural values.  2. Avoid Redundant or Overly Long Sentences  Problem: There are several sentences that are long and contain more than one idea.  Solution: Break them into several sentences to make them clearer and more academic.  Example: Global citizenship is not just a normative concept, but an operational framework that shapes students' active role in social and environmental issues.  3. Focus on Implications and Scientific Contributions  Problem: There is no specific paragraph that highlights the practical and theoretical implications of the findings.  Solution: Add a final paragraph that summarizes the contributions to literature, policy, or educational practice.  Example: This study contributes to the global citizenship literature by combining quantitative and qualitative approaches in the context of international students. The implications are important for higher education curriculum designers who want to instill global citizenship values more holistically.  4. Avoid Abrupt Topic Shifts  Problem: Suddenly entering the G20 and the Global Peace Index without a strong transition.  Solution: Connect the global data with the findings more systematically.  Example: The current global context, such as the decline in the world peace index by 0.56% in 2024, shows the urgency of integrating global citizenship in education. The theme of the G20 Brazil 2024 also emphasizes that global citizenship education is the foundation for social resilience and sustainable development.  5. End with a Strong and Reflective Sentence  Problem: The final sentence is too normative.  Solution: Close with a reflective sentence and based on the contribution of this study.  Example: Thus, the integration of global citizenship values in higher education plays a strategic role in creating a generation that is not only locally aware, but also globally responsible. | We have revised the conclusions to highlight the contrast between quantitative and qualitative findings more precisely.  We restructured several lengthy sentences in the Conclusion section. Ideas were separated into clearer academic sentences.  We have added a distinct concluding paragraph summarizing theoretical and practical implications.  We restructured the paragraph to establish a logical transition between the research findings and the global context. |
| **Note:** Thank you for your valuable comments. We tried to make all your suggestions, and we hope they meet your expectations. | |

| **REFEREE 4** | **AUTHORS** |
| --- | --- |
| Reviewer #4: The manuscript addresses a relevant topic concerning the global citizenship tendencies of international university students and their implications for sustainability. The use of a mixed-methods approach is appropriate and well-suited to the study objectives. The quantitative and qualitative data collected are generally sufficient and the conclusions drawn are aligned with the data presented. However, there are several areas that require revision before the manuscript is suitable for publication:  Abstract:  The abstract should be rewritten for clarity and conciseness. It currently lacks a clear statement of aims and does not adequately summarize the methods and key findings. Please include specific numerical or thematic results to reflect the study's contributions.  Introduction:  The rationale for conducting this study needs to be better articulated. A deeper engagement with relevant recent literature (especially post-2020) would strengthen the context. Consider citing more sources to support claims regarding the roles of international students in sustainability discourse.  Methods:  While the methods section outlines the study design, more detail is required on the sampling strategy, participant selection criteria, and ethical considerations. The explanation of the statistical analysis lacks clarity regarding assumptions checks and rationale for selecting non-parametric tests in certain cases.  Results:  Statistical results should be reported more consistently. For example, effect sizes are missing in some cases where p-values are provided. Consider using visual aids such as graphs or summarized tables to improve data presentation.  Discussion:  Several interpretations, particularly those linking qualitative findings to broader implications, appear speculative and should be revised to align more closely with the data. A discussion of the study’s limitations—such as representativeness, sample size, or cultural variability—is necessary.  Language and Style:  While generally intelligible, the manuscript contains numerous grammatical issues and long, complex sentences. A thorough language review is recommended to enhance readability and flow.  Once these revisions are addressed, the manuscript would make a valuable contribution to the literature on global citizenship and sustainability in higher education. | The revised version now clearly states the aim of the study, the research design (mixed method: convergent parallel), the number of participants (n=634 for the quantitative phase and n=37 for the qualitative phase), and specific thematic and statistical findings. We also clarified that the participants are international students studying at universities in Turkey. Furthermore, we now include specific results such as “moderate levels of global citizenship awareness in quantitative data” and “multifaceted evaluations in qualitative interviews,” which highlight the contributions of the study.  The Introduction section has been enriched by expanding the rationale with a more focused problem statement emphasizing the importance of understanding international students' roles in promoting global citizenship and sustainability. We have included recent literature from 2021–2024 (e.g., Liu et al., 2023; Cleofas & Labayo, 2022; Maguth & Yamaguchi, 2024) to reflect contemporary discourse on these themes.  We have expanded the Methods section to explicitly describe the sampling strategy for both the quantitative and qualitative phases. Participant inclusion criteria are now defined (e.g., active enrollment, region of origin). We also added detailed information about ethical approval (granted by the [Name] Ethics Committee, approval no. X), participant consent, and confidentiality procedures.  Furthermore, we clarified the rationale for using non-parametric tests. Normality assumptions were tested using Shapiro-Wilk and Kolmogorov-Smirnov tests, which indicated non-normal distributions for some subscales. Accordingly, Mann–Whitney U and Kruskal–Wallis H tests were applied, and this reasoning is now explicitly stated in the methods.  The Results section has been revised to ensure consistency in statistical reporting. We added a separate Limitations and Recommendations section. We reduced sentence length, minimized passive constructions, and adopted more concise academic language throughout. |
| **Note:** Thank you for your valuable comments. We tried to make all your suggestions, and we hope they meet your expectations. | |

| **REFEREE 5** | **AUTHORS** |
| --- | --- |
| Reviewer #5: The manuscript meets with the requirements with some adjustments and recommendations. Title: “Determining the Global Citizenship Tendencies of International University Students for a Sustainable World”  This manuscript explores an important and timely topic, how international students perceive and embody global citizenship, and how this relates to sustainable development. The mixed-methods design and sizeable sample strengthen the study. However, several issues related to theoretical framing, integration of findings, and data interpretation limit its contribution. Below are specific comments organized by theme.  In terms of novelty and contribution, the study topic is relevant, but the manuscript lacks a clearly defined research gap. The literature review broadly summarizes existing work but does not convincingly position this study as offering new insights. Therefore, it is suggested to clearly state how this study builds upon or differs from prior work, especially those using the Global Citizenship Scale or examining international students. Concerning theoretical framework, the manuscript lacks critical engagement with existing theories of global citizenship. There is limited reference to alternative or critical perspectives (e.g., postcolonial critiques, tensions with national identity). Therefore, it is recommended to consider integrating deeper theoretical reflections—what kind of global citizenship is being measured or promoted? What assumptions underline this framing? In the area of research method, Mixed-Methods Design applied while the convergent parallel design is appropriate, the manuscript does not sufficiently describe how the quantitative and qualitative data complement or contrast each other. However, it is suggested to add a section explicitly discussing how integration of the two data strands informs or refines your conclusions. Additionally, qualitative analysis- the qualitative component is underdeveloped. Themes are summarized broadly, and few participant quotes are included. Therefore, it is suggested to expand the thematic analysis with more illustrative quotations and deeper interpretive commentary by showing how students’ views vary or contradict one another.  Further, the interpretation of quantitative results tends to overstate the strength of findings, especially given that most effect sizes are small. It is better to align the tone of the discussion with the strength of the evidence. Use more cautious and precise language. Some statistically non-significant findings are discussed without adequate explanation to clearly distinguish between significant and non-significant results and avoid implying meaning where statistical support is lacking. Additionally, in the issue of contextualization, the study is conducted in Turkey, yet the potential influence of this specific context on international students’ experiences is not addressed. The discussion on how cultural, political, or institutional characteristics of Turkey may influence the findings are highly recommended. Meanwhile, the current data availability statement restricts access based on ethical concerns considering whether anonymized data could be shared or provide a stronger justification for restrictions to comply with PLOS ONE policies. In terms of writing and structure, the manuscript is generally well-written but contains some redundancy, particularly in descriptions of the Global Citizenship Scale and its sub-dimensions. It is suggested to revise for conciseness and avoid repetition. The use tables are clear and informative, but some captions could be more descriptive for standalone readability. Therefore, it is suggested to briefly summarize key takeaways in each caption. Methodological Clarification in more detail is needed regarding the rationale for selecting non-parametric tests in some cases. Also, it is better to clearly explain why specific tests were chosen and how assumptions of normality were addressed. Finally, it is also suggested to revise some grammatical errors that occurred in the manuscript.  Minor Revision: The paper has clear potential, but revisions are needed to enhance its theoretical depth, integrate findings more rigorously, and clarify interpretations. | we revised the final paragraph of the Introduction to clearly define the gap in the literature,  We have added a paragraph at the end of the literature review section clarifying how this study extends prior work.  The differences with the 3 studies used in the literature are added in the conclusion section where the quantitative findings are summarized.  In the Discussion section, we now reflect more critically on the type of global citizenship being assessed (cosmopolitan/liberal orientation) and acknowledge limitations in framing.  We revised the “Results” and “Discussion” sections to include a more explicit explanation of how the qualitative findings confirmed, complemented, or nuanced the quantitative data.  We revisited the qualitative analysis section.  We reviewed all the non-significant results and revised the discussion accordingly. Any speculative language implying meaning where no statistical support exists has been removed or reframed.  We expanded the Introduction and Discussion sections to consider the unique cultural, institutional, and political context of Turkey and how it may shape international students' experiences and perceptions.  We revised the Data Availability Statement to better align with PLOS ONE's policies. While participant confidentiality remains a priority, we now provide a stronger justification and indicate that anonymized datasets can be made available upon request to corresponding author.  We streamlined the description of the Global Citizenship Scale in the Method section to remove redundancies and enhance readability.  We revised the captions of all tables to include concise summaries of key findings, ensuring clarity for readers who may not read the full text in detail.  In the revised Method section, we have clearly explained the statistical assumptions tested and provided the rationale for choosing non-parametric tests where appropriate.  We carefully edited the manuscript for grammar, clarity, and style throughout. |
| **Note:** Thank you very much for your detailed suggestions. Thanks to your suggestions, our article will be more qualitative. We have tried to implement all your suggestions and hope that they meet your expectations. | |
