# Supplementary material for: Determining the global citizenship tendencies of international university students for a sustainable world
Source: PLoS One. 2025 Oct 31;20(10):e0335817. doi: 10.1371/journal.pone.0335817 (PMC12578196; doi:10.1371/journal.pone.0335817)
Supplement: Attachment — Submitted filename: Revision Text.docx [file pone.0335817.s006.docx]

**Abstract Revision Notes**

1. Clarity of the problem/research gap

The abstract goes straight to the objective without mentioning why it matters or what the previous research gap is. International journals usually require an opening sentence or two that emphasizes the urgency/major topic and the research gap.

2. More concise methodological details

The methods section is somewhat lengthy. International abstracts typically condense: mention the design, sample size, and instruments, but avoid technical details like "using a protocol developed by the researchers." This is more appropriate for a methods article, not an abstract.

3. Consistency of results

There is little repetition between quantitative and qualitative results. These can be summarized to key insights and then emphasize the convergence, as this is a mixed methods study.

4. International academic language

Some phrases are still long and could be condensed for greater efficiency. For example:

a. Quantitative findings indicate a moderate level of global citizenship awareness among university students, could be condensed: Quantitative findings revealed a moderate level of global citizenship awareness.
b. Many participants reported applying global citizenship principles in their daily routines, Many participants reported applying global citizenship principles in daily practices.
5. Closing abstract

The final section mentions policy recommendations, but they are still generic. It should be more specific: for example, integrating global citizenship and sustainability education into the higher education curriculum is recommended to strengthen students’ role as agents of sustainable change.

So, the main things that need to be revised are:

1. Add context to the problem/gap at the beginning.
2. Summarize the methodology section (without technical details).
3. Condense and emphasize the novelty of the results.
4. Use shorter, more direct, and formal academic language.
5. Strengthen the takeaways in the closing section.

**Keyword Revision Notes**

**keyword suggestions:**

Global Citizenship; Sustainability; International Students; Higher Education; Education for Sustainable Development (ESD).

**Introduction Revision Notes**

1. Logical Flow Structure

The current introduction feels long and repetitive (for example, the sections explaining "global citizenship" and "sustainable development" are explained more than once).

2. Clarity of Research Gap
a. Currently, the introduction is mostly descriptive and normative (explaining the concepts of global citizenship and sustainability), but lacks clarity on the gaps in previous research it aims to address.
b. There should be a sentence like: "While previous studies have examined global citizenship awareness in different contexts [refs], limited attention has been given to international students in Turkey, particularly in relation to sustainability awareness."
3. Strong International References

Only a few citations appear at the end ([1,2]). International journals typically demand more citations from Scopus/Web of Science articles from the last 5–10 years, especially to support the claim: globalization, global citizenship, sustainability, higher education, international students.

4. Academic and Concise Language
a. Many sentences remain long and repetitive. For example:
b. “Global citizens value universal principles such as justice, human rights, cultural pluralism, and environmental awareness…” This could be condensed to “Global citizens value universal principles such as justice, human rights, cultural pluralism, and environmental awareness.”
c. Avoid repeating the phrases “global citizens” or “sustainable development” repeatedly.
5. The closing paragraph should be more explicit.
a. Currently, the research objective is stated, but it is still mixed with narrative.
b. It should be made explicit in one or two strong sentences: “Therefore, this study aims to determine the global citizenship tendencies of international students in Turkey and to explore how these tendencies are related to sustainability awareness.”

So, the most necessary revisions:

1. Condense the description of globalization and global citizenship (avoid repetition).
2. Add recent international citations to strengthen the framework.
3. Clearly identify the research gap (what has been studied and what is missing).
4. Refine the language to be more concise and formal.
5. Close the introduction with a clear statement of the research objective.

**Method Revision Notes**

1. The Method Structure Is Too Long and Mixed

The methods section is a mix of background, technical explanations of the instrument, procedures, and validity and reliability test results. This section should be separated.

2. Redundancy & Excessive Detail
a. For example, the description of the Global Citizenship Scale (22 items, factors, minimum-maximum scores, reliability, etc.) is too detailed. International articles should simply state who developed it, a brief structure, examples of dimensions, and reliability (Cronbach's α). Item details are better placed in an appendix.
b. The explanation of the interview procedures (schedule, WhatsApp, faculty location, etc.) is too technical. Generally, it should be sufficient: "Semi-structured interviews were conducted face-to-face, audio-recorded, and transcribed for analysis."
3. Clarity of Sample Selection
a. You've mentioned the number of participants, their region of origin, and inclusion criteria, good
b. However, information about the sampling method (purposive, convenience, random?) is needed.
c. In international journals, details on how to contact students via email/WhatsApp are too operational → better summarized.
4. Research Ethics
a. You've mentioned ethics approval (good!).
b. However, it's best to present it concisely: the ethics approval number, the licensing body, and a statement that informed consent was obtained. No need for technical details like "Google Forms asks 'Are you willing to...'".
5. Data Analysis
a. For quantitative research: too detailed explanations of normal distribution theory (skewness, kurtosis, Shapiro-Wilk test with p<0.05, etc.) can be summarized. Sufficient: “Normality assumptions were tested (skewness, kurtosis, and Shapiro-Wilk test), and as some variables were non-normally distributed, non-parametric analyses (Spearman correlation, Mann–Whitney U, Kruskal-Wallis) were conducted.”
b. For qualitative research: the explanation is good (MAXQDA, thematic analysis, constant comparison). However, it can be summarized without the step-by-step WhatsApp follow-up with participants.
6. Language & Focus
a. Some sentences are still “stories” rather than “methods reports.” For example: “Students were reminded to complete the scale sent via Google Form before coming for the interview.” Better written formally: “Participants completed the scale prior to the interview sessions.”
b. Avoid first person (“the researcher conducted…”); use the formal passive voice.

So, the main revisions needed are:

1. Divide the methods into standard subsections.
2. Concise the description of the measurement tools and procedures (leave the rest in the appendix).
3. Emphasize the type of mixed methods design and the rationale for its selection, but avoid overly narrative.
4. Concise the data analysis section (don't go into too much detail about statistical theory).
5. Strengthen the explanation of sampling (what type).
6. Make the language more formal and less "chronological."

**limitations and implications Revision Notes**

1. A clearer separation between limitations and recommendations.

Currently, limitations and recommendations are intertwined. For example, the sentence "Furthermore, although qualitative data reveals that students experience conflict between global and national identities, this important information cannot be examined in depth" is a limitation. But immediately after that, a recommendation is presented, making it difficult for the reader to distinguish between them.

2. Avoid repetition of ideas.
a. The idea "the need for students from diverse geographic regions" appears several times with different wordings. It could be condensed into one clear limitation.
b. Similarly, "conflict between global and national identities" is mentioned more than once. This could be summarized to make it more powerful.
3. Make limitations more concise and specific. Examples of revised wording:
a. Sample limitations (only students from certain regions, no representation of Europe, Australia, or developed Asian countries).
b. Data triangulation limitations (quantitative results cannot be confirmed with qualitative ones due to limited participants).
c. Issue exploration limitations (global vs. national identity conflict is not explored in more depth).
4. Make recommendations more structured.
a. Methodological recommendations: cross-national studies, involving students from more backgrounds, mixed-methods with a more balanced design.
b. Thematic recommendations: in-depth exploration of global versus national identity.
c. Practical recommendations: development of educational interventions, integration of global citizenship content into the curriculum, utilization of social media.
5. Avoid an overly normative or “long-winded” tone.

The section on “creating a more conscious global society in the long term” is more appropriate in the Discussion or Conclusion, not in the limitations/recommendations section.

6. More concise academic language. If you want an international level, use more concise wording, for example:

A major limitation of this study is the restricted diversity of qualitative participants, which excluded students from Europe, Australia, and developed Asian countries. Future research should address this gap through comparative cross-regional interviews to triangulate quantitative findings and deepen the exploration of identity conflicts between global and national citizenship.”

So the revisions are primarily:

1. separate the limitations and recommendations sections more clearly,
2. avoid repetition,
3. condense language,
4. focus on points directly relevant to the study.

**Conclusion and Discussion Revision Notes**

1. Mixed Discussion and Conclusions
a. Currently, the "Conclusions and Discussion" section is too long, with details like results and discussion.
b. Generally, the Discussion section is used to compare findings with the literature, provide interpretations, and explain implications.
c. The Conclusion should be concise, containing only answers to the research questions, main contributions, limitations, and suggestions for further research.
2. Excessive Repetition

Many points are repeated, for example, about moderate scores on global citizenship, the impact of social media, or regional differences. These points should be mentioned once to strengthen the argument.

3. Lack of Focus on Novelty
a. Currently, the findings are predominantly descriptive ("medium scores," "Europe is higher," "social media is influential"). It's important to emphasize what's new compared to previous research.
b. For example: is this study stronger because it compares multiple regions simultaneously? Or because it includes social media and global participation variables simultaneously?
4. Practical and theoretical implications haven't been separated.

Could you clarify: what contribution do these findings make to global citizenship theory? What are the implications for university or international institution policy?

5. The conclusion is too long
a. The conclusion should be brief (1–3 paragraphs), without further literature citations, and without repeating the results in detail.
b. Simply formulate the main findings, research contributions, limitations, and recommendations for further research.
6. International academic language
a. Some sentences are still long narratives. For international journals, it's best to condense them, using straightforward sentences, for example:
b. "The findings reveal that international students generally demonstrate a moderate level of global citizenship, with significant regional and experiential variations."
c. Then go straight to the interpretation "This suggests that while awareness exists, translating it into sustained responsibility and action remains limited."

So the revisions should be roughly:

1. Separate the Discussion (interpretation + comparison with the literature + implications) and the Conclusion (short answer, contributions, recommendations).
2. Concise the Conclusion section, removing detailed figures/variables already included in the results.
3. Clarify the novelty (what this study adds to the literature).
4. Add limitations and recommendations for further research (e.g. limitations of the Turkish context, predominance of respondents from certain regions, or limitations of the instrument).
