# Supplementary material for: Determining the global citizenship tendencies of international university students for a sustainable world
Source: PLoS One. 2025 Oct 31;20(10):e0335817. doi: 10.1371/journal.pone.0335817 (PMC12578196; doi:10.1371/journal.pone.0335817)
Supplement: Attachment — Submitted filename: Review by Uzlifatul Masruroh Isnawati.docx [file pone.0335817.s007.docx]

Review of the manuscript **“Determining the Global Citizenship Tendencies of International University Students for a Sustainable World” (PONE-D-25-07333R1)**

**Strengths**

1. **Relevance & Timeliness**
   The topic—linking global citizenship with sustainability among international students—is significant and aligns well with contemporary global education and sustainability debates.
2. **Mixed-Methods Design**
   The convergent parallel mixed-methods approach (quantitative survey + qualitative interviews) is a strong design choice. It allows for triangulation and enriches the findings.
3. **Sample Size & Diversity**
   With 634 quantitative participants and 37 qualitative interviews, the dataset is fairly robust for this type of research.
4. **Revisions Addressed Reviewers’ Concerns**
   The authors have made extensive revisions: restructuring the introduction, clarifying methods, improving conclusions, adding effect sizes, and refining language.
5. **Policy Implications**
   The paper concludes with useful recommendations for integrating global citizenship and sustainability into higher education curricula.

**Weaknesses & Areas for Improvement**

1. **Abstract**  is still slightly long and descriptive. While improved, it could be more concise by emphasizing *novel contributions* and highlighting *specific results* (e.g., effect sizes, strongest themes).
2. **Introduction** is much stronger after revisions, but it remains somewhat heavy on general globalization theory. Further trimming could maintain focus on the study’s contribution. The research gap is now stated, but could be *more sharply articulated*: How exactly does this study advance beyond previous Global Citizenship Scale applications?
3. **Theoretical Framework** is limited engagement with critical or alternative views of global citizenship (e.g., postcolonial, Southern perspectives). Adding these would strengthen the study’s conceptual depth.
4. **Methods** are well clarified, the rationale for using *English-only participants* could raise selection bias concerns (students with weaker English skills may have been excluded, reducing representativeness).

The description of *trustworthiness procedures* is adequate but could cite more seminal qualitative methodology sources.

5. **Results** can be seen from the tables are comprehensive, but some remain dense. Consider condensing or using visuals (e.g., charts for regional comparisons). The quantitative findings focus on statistical significance but give less attention to *practical significance* (effect sizes are small, which should be acknowledged).
6. **Qualitative Analysis** is improved, but still somewhat underdeveloped. More student quotations and contrasting voices could better demonstrate diversity in perspectives.
7. **Discussion** states some claims still lean toward speculation, especially when connecting findings to broader global trends. The Turkish context is mentioned but could be more deeply integrated (e.g., how local policies or cultural factors shape students’ views).
8. **Conclusion** is stronger than before, but the final reflection could emphasize **specific contributions to theory and practice** more clearly. Future research directions could be better detailed (e.g., longitudinal studies, intercultural comparisons).
9. **Language & Style** improved but still wordy in parts. Some sentences remain long and complex—further editing for clarity and conciseness is recommended.

**Overall Evaluation**

**Suitability for PLOS ONE**: The article now aligns more closely with PLOS ONE’s requirements for methodological clarity, data availability, and policy relevance. **Contribution**: Moderate to strong. The study provides empirical insights into how international students in Turkey engage with global citizenship and sustainability, though theoretical framing could be richer. **Recommendation**: **Accept with minor revisions** Tighten the abstract and introduction. Add more nuance to the theoretical framework. Enrich qualitative results with additional quotes. Streamline results presentation for readability.

**General Evaluation**

This manuscript addresses a timely and relevant issue, examining how international university students in Turkey perceive and enact global citizenship in relation to sustainability. The use of a convergent parallel mixed-methods design strengthens the study, and the revisions made in response to prior reviewer feedback have clearly improved the structure, clarity, and scholarly contribution of the paper. Overall, the manuscript is suitable for publication in *PLOS ONE*, but I recommend **minor revisions** to further refine the clarity, theoretical framing, and presentation of findings. However, this manuscript is well-prepared and contributes meaningful insights into the role of international students in advancing global citizenship and sustainability. With further tightening of the abstract, enrichment of theoretical framing, and clearer integration of qualitative findings, the article will make a valuable contribution to the literature.
