# Supplementary material for: Determining the global citizenship tendencies of international university students for a sustainable world
Source: PLoS One. 2025 Oct 31;20(10):e0335817. doi: 10.1371/journal.pone.0335817 (PMC12578196; doi:10.1371/journal.pone.0335817)
Supplement: Attachment — Submitted filename: Response to Reviewers.docx [file pone.0335817.s008.docx]

| **Determining The Global Citizenship Tendencies of International University Students for a Sustainable World**  **Referee Recommendations and Author Disclosures** | |
| --- | --- |
| **SUMMARY** | |
| **REFEREE 3** | **Authors** |
| The summary goes straight to the purpose; it does not state why it is important or what the gap in previous research is. | A few introductory sentences have been added to highlight the research gap and the importance of the topic. |
| The method section is a bit long | Technical details such as "using a protocol developed by researchers" were omitted |
| There is little duplication between the quantitative and qualitative results. These should be summarized as key findings, then the mixed-methods nature of the study should be emphasized. | Increased emphasis on mixed methods. |
| Some phrases are still long; they can be written more briefly and directly. | Edited as you suggested. |
| The final section includes policy recommendations, but they are still general. | Edited as you suggested. |
| Suggested keywords: Global Citizenship; Sustainability; International Students; Higher Education; Education for Sustainable Development. | The keywords have been edited according to your suggestion. |
| **INTRODUCTION** | |
| The current introduction is long and repetitive. The concepts of “global citizenship” and “sustainable development” are introduced multiple times. | Some sections were removed to eliminate the excessive explanations you mentioned. |
| The introduction is rather descriptive and normative, but there is no clear emphasis on shortcomings in previous studies.  While previous studies have examined global citizenship awareness in different contexts [ refs ], its relationship with sustainability awareness among international students in Turkey has not received sufficient focus. ” | Added to the introduction. |
| expect more citations from Scopus /Web of Science articles from the last 5–10 years, especially on concepts such as globalization, global citizenship, sustainability, higher education and international students . | In the introduction, we wanted to present the problem statement in our own words. However, different references were added in the section where the concepts were explained. |
| Some sentences are still long and repetitive.  “Global citizens value universal principles such as justice, human rights, cultural pluralism, and environmental awareness…” → “Global citizens value universal principles such as justice, human rights, cultural pluralism, and environmental awareness.” | It has been revised according to your suggestion. |
| Some sentences are still long and repetitive. | Efforts were made to eliminate repetitions. |
| Now the purpose of the research is explained, but it gets lost in the narrative. | In line with your suggestion, the following sentence was added: "This study aims to determine the global citizenship tendencies of international students in Turkey and to reveal the relationship between these tendencies and sustainability awareness." |
| **METHOD** | |
| Background, technical details of instruments, procedures, and validity and reliability results are intertwined. They should be divided into separate subheadings. | In line with your suggestion, clear sentences were used under the same headings. |
| Global Citizenship Scale (22 items, factors, min-max , etc.) is too detailed for a summary. Simply providing the developer, general structure, dimension examples, and reliability value ( Cronbach's α) is sufficient. Details should be included in the appendix . | It has been reviewed according to your suggestion. |
| The way the interviews were conducted (WhatsApp, faculty room, etc.) was very operational. In short: " Semi-structured interviews were conducted face-to-face, audio-recorded, and transcribed. " | This suggestion has been added to the text verbatim. |
| The sampling method (purposive, convenience sample, random?) should be specified. | The sampling method has been added. |
| Reaching students via email/WhatsApp is too operational → should be summarized. | An attempt was made to edit these sections. |
| Research ethics approval number, institution, and where informed consent was obtained. Details such as “Google Form question: Do you agree to participate?” are unnecessary. | Some of these details were removed and ethical approval was obtained where appropriate. |
| Quantitative analysis: Too much emphasis is placed on normal distribution theory. Sufficient: “Assumptions of normality were tested, and since some variables were not normally distributed, nonparametric tests ( Spearman correlation, Mann–Whitney U, Kruskal -Wallis) were used.” | Edited according to your suggestion. |
| Qualitative analysis: MAXQDA, thematic analysis, and comparative approaches are appropriate. However, details like WhatsApp tracking should be omitted. | Edited according to your suggestion. |
| Passive voice should be used instead of first person singular. | Language check was done again. |
| **Limitations and Recommendations** | |
| Limitations and recommendations must be separated. They are currently intertwined. | It was divided into two parts. |
| Avoid repetition. For example, "students from different geographic areas should participate" appears several times with different wording. | Efforts were made to eliminate repetitions. |
| Limitations should be written more clearly and specifically.  a. Sample limitation: No students from Europe, Australia, or developed Asian countries.  b. Data triangulation limitation: Quantitative data cannot be fully validated due to the small number of qualitative participants.  c. Topic limitation: Global–national identity conflict could not be examined in more depth. | The limitation has been adjusted. |
| Suggestions should be structured  a. Methodological: larger, comparative studies.  b. Thematic: in-depth examination of the global–national identity conflict.  c. Practical: educational interventions, integration into the curriculum, use of social media. | Edited in the flow you suggested. |
| Normative and long narrative should be avoided.  Sentences like “creating a conscious global society in the long term” belong in the discussion/conclusion section. | The sentences expressed have been changed. |
| More concise academic language.  For example : “An important limitation of this study is the limited diversity of qualitative participants; students from Europe, Australia, and developed Asian countries were excluded. Future research should triangulate the quantitative findings with comparative regional interviews and examine global–national civic identity conflicts more deeply.” | Language check was done. |
| **Discussion and Conclusion** | |
| The debate and results are mixed  Discussion should focus on literature comparison + interpretation + inferences.  The conclusion should be brief: answers to research questions, contributions, limitations, recommendations. | Discussion has been edited. Conclusion has been edited. Limitations and recommendations have been presented under separate headings. |
| **Too much repetition**  Points like mid-range scores, social media impact, regional differences are recurring. | Repetitions reviewed |
| **Insufficient emphasis on innovation**  It's unclear what the study adds to the literature. Is it a multi-region comparison or a social media variable? These should be highlighted. | The conclusion was tried to be given at the end of the section. |
| **Practical and theoretical contributions should be separated**  The contribution of the findings to theory and practical implications at the university/policy level should be clear. |  |
| **The result is too long**  a. The conclusion should be 1–3 paragraphs.  b. Detailed figures/analysis should not be repeated.  c. Main findings + contributions + limitations + recommendations should be summarized. | The result has been edited |
| **International academic language**  Instead of long sentences, use short and clear sentences:  For example : “The findings indicate that international students generally exhibit moderate levels of global citizenship, but there are regional and experiential differences. This suggests that awareness exists but its translation into sustainable responsibility remains limited.” | Sentences reviewed |
| Note: First of all, thank you very much for your detailed suggestions. We hope you like our edits.  **We attempted to incorporate the corrections made by all four reviewers during our review of our manuscript. If any corrections are missing or not to your liking, please take this into consideration.**  Your suggestions are valuable to us, our article will be more qualified with your suggestions. | |

| **REFEREE 5** | **AUTHORS** |
| --- | --- |
| Tighten the abstract and introduction. | Summary and introduction have been edited. |
| Add more nuance to the theoretical framework. | Added different and updated resources. |
| Enrich qualitative results with additional quotes. | It has been reviewed and increased. |
| Streamline results presentation for readability. | Reviewed and edited. |
| Note: Thank you for your suggestions and contributions to the article. We hope our edits meet your expectations. | |
